# Molecular docking and molecular dynamics investigation of novels thiazolopyrimidines binding to canonical and alternative allosteric sites of kinesin Eg5

**DOI:** 10.1007/s00894-026-06958-3

**Published:** 2026-09-15

**Authors:** Vinícius S. Cunha, Arlan S. Gonçalves, Reginaldo B. dos Santos, Ricardo M. Kuster

**Affiliations:** 1 Department of Chemistry, Federal University of Espírito Santo, Av. Fernando Ferrari, Vitória, Espírito Santo 29075-910 Brazil; 2 Department of Chemistry, Federal Institute of Education, Science and Technology of Espírito Santo, Av. Min. Salgado Filho, Vila Velha, Espírito Santo 29106-010 Brazil

**Keywords:** Eg5, Thiazolopyrimidines, Noncanonical allosteric site, Molecular docking, Molecular dynamics

## Abstract

**Context:**

Human kinesin Eg5 is essential for bipolar spindle assembly, and most allosteric inhibitors bind to the canonical pocket formed by the α2 helix, loop 5, and the α3 helix. Eleven thiazolo[3,2-*a*]pyrimidinethiones were evaluated as ligands for two sites in Eg5: this canonical pocket and an alternative region near loop 8. All derivatives showed favorable docking poses at both sites, with average energies of -7.95 and -7.79 kcal.mol^−1^ for the loop 5 and loop 8 sites, respectively, whereas monastrol favored loop 5 by 1.6 kcal.mol^−1^. Compounds **1a** and **2d** remained associated with both sites during 500 ns simulations, with ligand root-mean-square deviations between 1.00 and 1.53 Å. MM-PBSA estimates ranged from -26.02 to -22.92 kcal.mol^−1^, compared with -22.14 kcal.mol^−1^ for monastrol. RMSF calculations revealed site- and ligand-dependent redistribution of mobility in loop 5, loop 8, Switch I, Switch II, the microtubule-binding site, the cover-neck bundle, and the neck linker. These results indicate that this scaffold is promising for the exploration of alternative allosteric sites in Eg5, although experimental confirmation is required.

**Method:**

The Switch II segment was reconstructed with SWISS-MODEL and evaluated with PDBsum. The structures were prepared with PDB2PQR, AutoDockTools, and Open Babel. Redocking compared AutoDock Vina, QuickVina 2, and QuickVina-W, and QuickVina-W with exhaustiveness 32 was selected. Six systems were simulated for 500 ns in GROMACS using the OPLS-AA/L force field, TIP3P water, and ligand parameters obtained with ACPYPE. Visual Molecular Dynamics was used for trajectory measurements, Discovery Studio Visualizer for interaction analysis, and g_mmpbsa for binding-energy estimation and per-residue decomposition.

**Supplementary information:**

The online version contains supplementary material available at https://doi.org/10.1007/s00894-026-06958-3.

## Introduction

Cancer is one of the main obstacles to increasing life expectancy and remains among the leading causes of death worldwide. According to the Global Cancer Observatory, 19.3 million new cases and nearly 10 million cancer deaths were estimated in 2020. In 2019, the disease also ranked first or second among the causes of death before the age of 70 in 112 of 183 countries [1]. One strategy to control its progression is to develop antiproliferative compounds that act on processes essential to cell division. In this regard, the mitotic spindle is a validated target because its formation is indispensable for chromosome segregation. Taxanes and vinca alkaloids have been used for this purpose, interfering with tubulin and microtubule dynamics. However, because microtubules also function in nonproliferative cells, these drugs can cause neurotoxicity, resistance, and other dose-limiting effects [2]. Therefore, more selective mitotic targets must be studied.

**Fig. 1:**
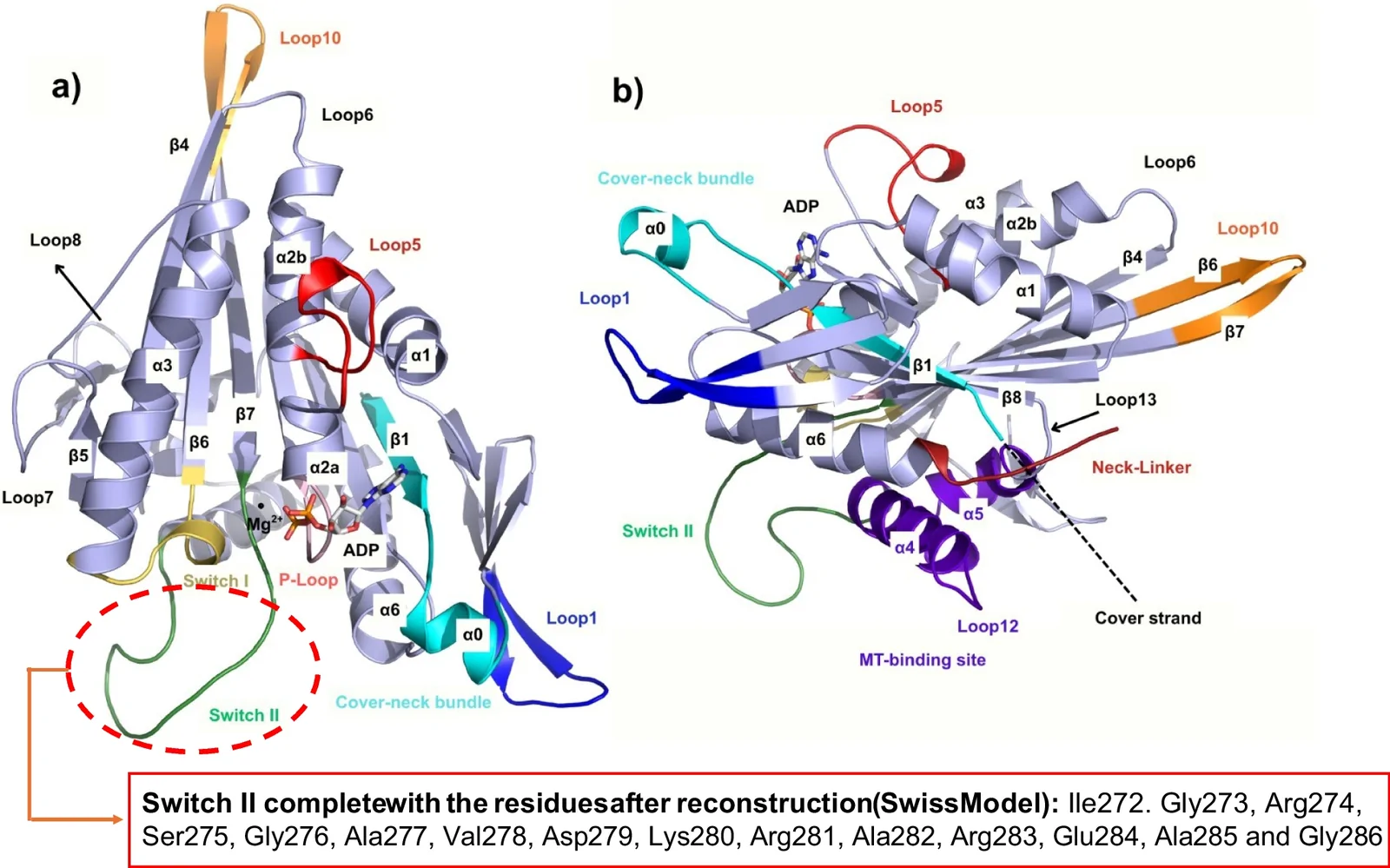
Structure of the Eg5 motor domain. **a**) Top-down view; **b**) Side view

In this context, a good candidate is the Eg5 protein, also known as KIF11 or kinesin spindle protein (KSP), a motor protein of the kinesin family involved in mitosis (Fig. 1). Through its motor domain, Eg5 uses the energy from ATP hydrolysis to perform mechanical work. Organized as a homotetramer, it crosslinks antiparallel microtubules and promotes their sliding in opposite directions, generating the forces required to separate centrosomes and form the bipolar spindle [2, 3]. Its inhibition impairs pole separation, produces monopolar spindles, and keeps the cell arrested in mitosis, potentially leading to apoptosis [2–4]. Studies with the MCF-7, AGS, and HCT116 cell lines reinforce the interest in this target [5–7], while its overexpression in breast, gastric, and colorectal tumors has been associated with progression or unfavorable prognoses [3, 6, 8]. Thus, Eg5 represents a validated antitumor target that is potentially more specific than tubulin.

The first selective Eg5 inhibitor was monastrol, identified in 1999 by Mayer and colleagues in a phenotypic screen [9]. It binds to an allosteric cavity located approximately 10 Å from the active site and formed mainly by the α2 helix, Loop5, and the α3 helix. This binding reorganizes Loop5 and reduces ADP release, impairing the protein's mechanochemical cycle without competing directly with ATP [4, 10]. As a result, the cell develops the characteristic monoastral phenotype. However, the relatively low potency of monastrol limited its therapeutic application and stimulated the search for more efficient inhibitors.

Among the compounds developed subsequently, ispinesib showed high potency in vitro and advanced to phase I and II clinical trials. Structural studies confirmed its binding to the same α2/L5/α3 pocket and stabilization of the Eg5-ADP complex [10, 11]. In clinical trials, however, efficacy was variable, and prolonged or febrile neutropenia constituted a dose-limiting toxicity [12–14]. Filanesib, or ARRY-520, is another nanomolar inhibitor of this site and showed relevant results in hematological malignancies, such as multiple myeloma [12, 15]. In 2011, AZD4877 was described as a thiazolopyrimidine clinical candidate related to ispinesib. This compound inhibits Eg5 ATPase, induces monoasters, and showed preclinical antitumor activity, although its clinical efficacy as monotherapy was limited [4, 16].

Despite the structural differences among monastrol, ispinesib, filanesib, and AZD4877, most known inhibitors act at the allosteric pocket associated with Loop5. The compounds STLC, K858, MK-0731, and SB-743921 also recognize this region [12, 15]. The concentration of studies on a single site clarified its allosteric mechanism but revealed limitations. Mutations in the α2/L5/α3 pocket can reduce the affinity of different inhibitors without impairing the catalytic function of the protein, favoring resistance [5, 15]. Therefore, exploring other regions of Eg5 may expand the chemical space and reveal distinct inhibition mechanisms.

Biaryl inhibitors of the GSK series bind to the pocket between the α4 and α6 helices, near the microtubule-interaction region, and are competitive with ATP [12, 15]. Zhang and colleagues also identified the S1 and S2 sites [5]. The S1 site is near the neck linker and Loop8, and the direct binding of SRI35566 to Eg5 was corroborated by STD-NMR. This compound and its analogs SRI35565 and SRI35564 inhibited ATPase, increased monopolar spindle formation, and reduced the viability of HCT116 cells [5]. Curcumin binding was proposed for a region involving Loop8, β4, β5, and β6; subsequent calculations indicated greater compatibility with the α4/α6 pocket and highlighted the need for experimental validation [15]. Thus, the safest conclusion is that potentially exploitable alternative regions exist in Eg5, but they remain poorly investigated and few ligands have been evaluated in these environments.

Thiazolo[3,2-*a*]pyrimidines are fused heterocyclic systems with structural diversity and known antitumor potential [17, 18]. AZD4877 demonstrates that a thiazolopyrimidine core can be compatible with Eg5 inhibition [16]. Other derivatives of the class showed cytotoxicity, cell-cycle alterations, and induction of apoptosis [18]. A series of novels thiazolopyrimidines was synthesized in our laboratory providing a suitable chemical set for investigating different regions of Eg5. Although numerous Eg5 inhibitors have been reported, most studies focus on the allosteric pocket associated with Loop5. The ability of thiazolopyrimidines to interact with alternative regions of Eg5, especially near Loop8, remains poorly explored. Therefore, this study aimed to investigate synthesized thiazolopyrimidines as potential Eg5 inhibitors through molecular docking and molecular dynamics simulations. Binding to the pocket associated with Loop5 and to an alternative region near Loop8 was evaluated, as were the consequences of these interactions for protein stability, flexibility, and dynamics To strengthen the connection between the synthetic and computational components of this study, the synthesized thiazolopyrimidines were treated as a chemically related series capable of recognizing two distinct allosteric environments within the same protein target. Accordingly, three hypotheses were tested: (i) thiazolopyrimidinethiones can bind not only to the classical loop 5 pocket but also to the alternative loop 8 region; (ii) the two binding modes exhibit comparable energetic stability; and (iii) occupation of the loop 8 pocket induces a distinct redistribution of Eg5 dynamics, consistent with an alternative mechanism of allosteric inhibition.

## Methods

### Chemicals and general experimental procedures

The reagents and solvents were purchased from commercial suppliers and used without prior treatment. Reagents used: choline chloride (Sigma-Aldrich, ≥ 98%), urea (Sigma-Aldrich, ≥ 99%), thiourea (Sigma-Aldrich, ≥ 99%), ethyl acetoacetate (Sigma-Aldrich, ≥ 99%), benzaldehyde (Sigma-Aldrich, ≥ 99%), 4-methoxybenzaldehyde (Sigma-Aldrich, ≥ 98%), 4-nitrobenzaldehyde (Sigma-Aldrich, ≥ 99%), 4-chlorobenzaldehyde (Sigma-Aldrich, ≥ 98%), 3-hydroxybenzaldehyde (Sigma-Aldrich, ≥ 98%), indole-3-carboxaldehyde (Sigma-Aldrich, ≥ 98%), chloroacetic acid (Sigma-Aldrich, ≥ 99%), sulfuric acid (Vetec, 95–98%), hydrochloric acid (Vetec, 37%).

Reaction progress was monitored by thin-layer chromatography (TLC) on UV254 silica-gel plates (250 µm), with visualization under UV light and, when necessary, staining with vanillin. Purifications were conducted by column chromatography on silica gel 60 (70–230 mesh), using appropriate solvent mixtures.

Melting points were determined for characterization purposes and are reported as the observed values. The values may differ from the literature because of differences in instrumentation, heating rate, sample preparation, and experimental conditions. FTIR spectra were obtained on a PerkinElmer Spectrum 400 spectrometer (ATR, ZnSe) over the range of 4000–400 cm^−1^. The ^1^H (400 MHz) and ^13^C (101 MHz) NMR spectra were recorded on a Varian spectrometer using CDCl_3_ or DMSO-d_6_ and TMS as the internal standard. Complete characterization data are presented in the Supplementary Material.

### Synthesis

**Fig. 2:**
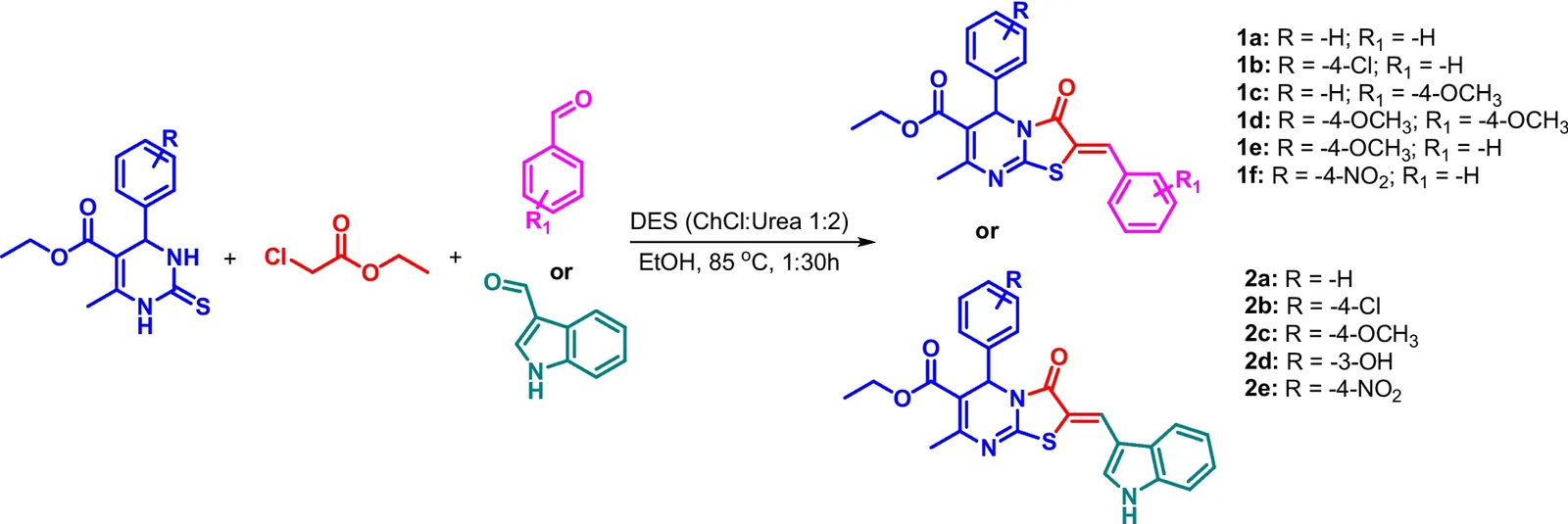
Reaction schemes for the synthesis of thiazolo[3,2-*a*]pyrimidines

The thiazolo[3,2-*a*]pyrimidines were obtained from the respective previously synthesized DHPMTs (dihydropyrimidinethiones) (Fig. 2). Choline chloride (1.0 mmol) and urea (2.0 mmol) were heated at 85 °C until the deep eutectic solvent (DES) formed. The corresponding DHPMT (1.0 mmol), ethyl chloroacetate (previously synthesized, 1.5 mmol), and absolute ethanol (3.0 mmol) were added, and the mixture was stirred for 30 min. The corresponding aldehyde (1.0 mmol) was then added, and the reaction was maintained at 85 °C for another 1 h. After the addition of absolute ethanol, the mixture was poured into ice-cold distilled water. The precipitate was filtered and dried, and washed with ethanol when necessary. Yields and complete characterization data are presented in the Supplementary Material. Thus, the synthetic campaign furnished the structurally related ligand set used in the subsequent comparative site analysis, directly linking compound preparation to the evaluation of canonical and alternative Eg5 allosteric recognition.

### Eg5 structure selection and preparation

The crystallographic structure of the human kinesin Eg5 motor domain complexed with Mg–ADP and monastrol was obtained from the Protein Data Bank under code 1X88. The structure was determined by X-ray diffraction at a resolution of 1.80 Å [19]. The asymmetric unit contains two motor-domain chains. Chain A was selected for the calculations. The second chain, crystallographic monastrol, and molecules considered nonessential were separated or removed using scripts run in a Linux environment. The ADP ligand and Mg^2+^ ion were retained in the protein structure.

The missing segment of the Switch II region was reconstructed by comparative modeling on the SWISS-MODEL server [20] (Fig. 1). The reconstructed segment comprised residues Ile272–Gly286: Ile272, Gly273, Arg274, Ser275, Gly276, Ala277, Val278, Asp279, Lys280, Arg281, Ala282, Arg283, Glu284, Ala285, and Gly286. The modeled structure showed a Ramachandran distribution comparable to the 1X88 template, with 93.6% of residues in the most favored regions, 6.1% in additionally allowed regions, and only 0.3% in disallowed regions [21]. The protonation states of the residues at pH 7.4 and the atomic parameters of the AMBER force field were assigned using the PDB2PQR server [22]. The final structure was converted to PDBQT format using AutoDock Tools 1.5.7. Polar hydrogens were added, partial charges were assigned by the Kollman method, and the atom types used by the docking program were defined.

### Docking studies

The three-dimensional geometries of compounds **1a-f** and **2a-e** were generated from the two-dimensional structures, adjusted for pH 7.4, and subjected to geometry optimization with Open Babel [23]. For redocking of crystallographic monastrol, AutoDock Vina, QuickVina 2, and QuickVina-W were compared while retaining 20 binding modes and testing exhaustiveness values of 8, 16, and 32 [24, 25]. The selected grid box has dimensions of 46 × 40 × 40 Å and is centered at coordinates x = 31.073, y = 31.557, and z = 103.733. This box simultaneously encompassed the classical allosteric site formed by the α2/loop 5/α3 region and the second pocket located on the opposite face of the motor domain, near loop 8. The predicted pose was compared with the crystallographic conformation, and the root-mean-square deviation (RMSD) was calculated in VMD [26].

The best result in reproducing the crystallographic pose and docking energy was obtained with QuickVina-W and exhaustiveness 32; these parameters were therefore used in the subsequent calculations. A total of 20 runs were performed for each ligand, generating up to 20 modes per run. The poses were classified according to their location at the loop 5 site or the loop 8 site, and the lowest-energy pose in each pocket was used to compare the compounds. The site referred to in this work as the “loop 8 site” corresponds topologically to the S1 allosteric site previously described for Eg5 [5]. The two-dimensional interaction maps were inspected in Discovery Studio Visualizer.

### Molecular dynamics simulations

Compounds **1a** and **2d** were selected as representatives of the two series because they showed favorable docking results at both sites and no energy difference between the pockets. Six systems were constructed: Eg5-ADP, Eg5-ADP-monastrol, Eg5-ADP-**1a** at the loop 5 site, Eg5-ADP-**1a** at the loop 8 site, Eg5-ADP-**2d** at the loop 5 site, and Eg5-ADP-**2d** at the loop 8 site.

The simulations were performed with the GROMACS package [27]. The protein was described by the OPLS-AA/L force field [28], and water was represented by the TIP3P model [29]. The ligands were protonated at pH 7.4, preoptimized with MMFF94, and parameterized with ACPYPE, with adaptation of the atom types to the OPLS-AA/L force field [30]. Each complex was centered in a dodecahedral box with a minimum distance of 1.5 nm between the system and the box edge, solvated, and neutralized with counterions.

For each system, a steepest-descent minimization with positional restraints, a steepest-descent minimization without restraints, and an additional minimization step using L-BFGS were performed. This was followed by a 500 ps equilibration step with positional restraints on the system. The temperature was maintained at 310 K by the velocity-rescale thermostat, and the pressure was isotropically coupled to 1 bar by the Berendsen barostat. Long-range electrostatic interactions were treated by the particle mesh Ewald method, with a cutoff radius of 1.0 nm; the same radius was applied to van der Waals interactions. All covalent bonds were constrained by the LINCS algorithm, allowing an integration time step of 2 fs.

The production trajectories were calculated for 500 ns without positional restraints, preserving the temperature, pressure, periodicity, and nonbonded-interaction treatment conditions used in equilibration. Coordinates were stored every 20 ps. They were corrected for periodic boundary conditions and least-squares fitted to the protein atoms. Global stability was evaluated by protein RMSD and the RMSD of each ligand after fitting to the ligand itself. The first 100 ns of system instability were disregarded for the mean RMSD calculations. The flexibility of the secondary structures was quantified by the per-residue root-mean-square fluctuation (RMSF). In addition to the complete profile, the cover-neck bundle (residues 25–40), loop 5 (residues 110–140), loop 8 (residues 186–194), Switch I (residues 226–236), motor end or motor tip (residues 236–266), Switch II (residues 268–288), microtubule-binding interface (residues 292–324), and neck linker (residues 356–364) functional regions were analyzed. Binding energies were estimated by MM-PBSA with the g_mmpbsa program [31]. Per-residue energy decomposition was also calculated.

Compaction of the Eg5 motor domain was evaluated by the radius of gyration (Rg), and solvent exposure was monitored by the solvent-accessible surface area (SASA), both calculated with the GROMACS package. The number of hydrogen bonds between the protein and the ligand was monitored over the simulation time, and donor-acceptor combinations were examined by their occupancies using VMD. The first 100 ns were disregarded for calculation of the average and standard deviation, consistently with the RMSD analysis. Protein-ligand contacts were defined by a maximum distance of 3.5 Å and represented in temporal maps with 5 ns windows; the trajectories were sampled every 0.2 ns to construct these maps.

## Results

### Docking studies

**Fig. 3:**
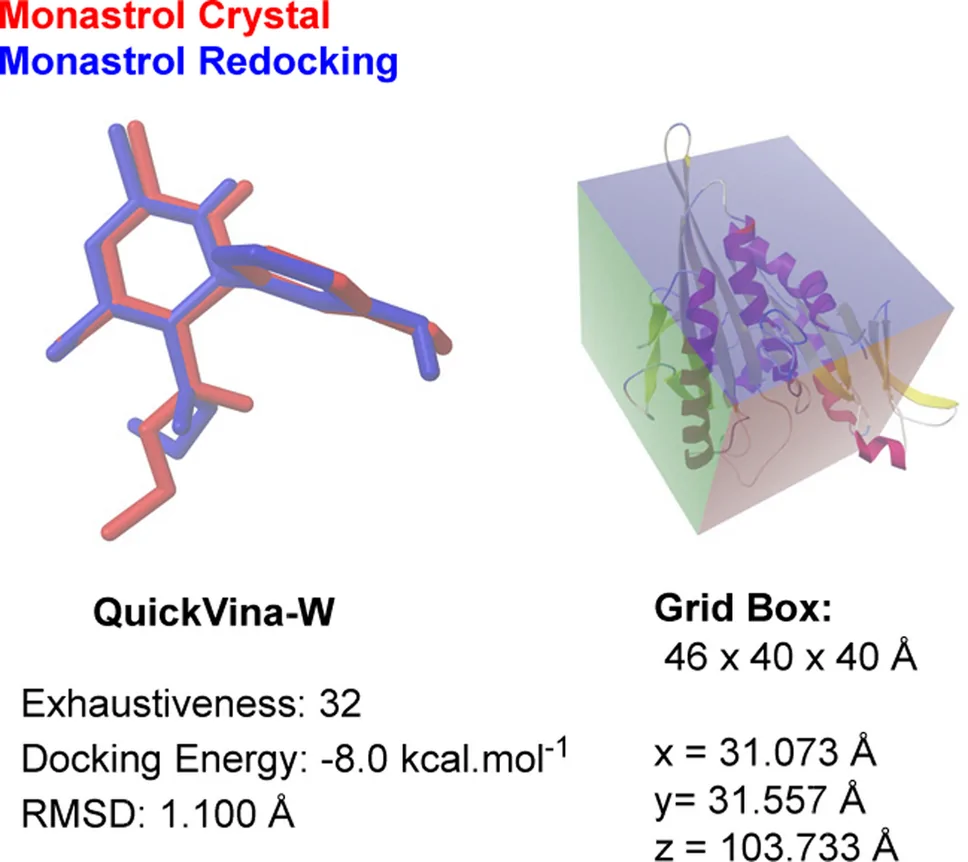
Superposition of crystallographic monastrol (red) and the best pose obtained in redocking (blue) with QuickVina-W

In redocking, QuickVina-W with exhaustiveness 32 reproduced the crystallographic orientation of monastrol with an RMSD of 1.10 Å and a docking energy of −8.0 kcal.mol^−1^ (Fig. 3). The overlap between the crystallographic and calculated poses preserved the general orientation of the dihydropyrimidinethione ring in the α2/loop 5/α3 pocket. This result, together with the RMSD below 2 Å, supported the use of the protocol to compare poses at the two allosteric sites.

**Fig. 4:**
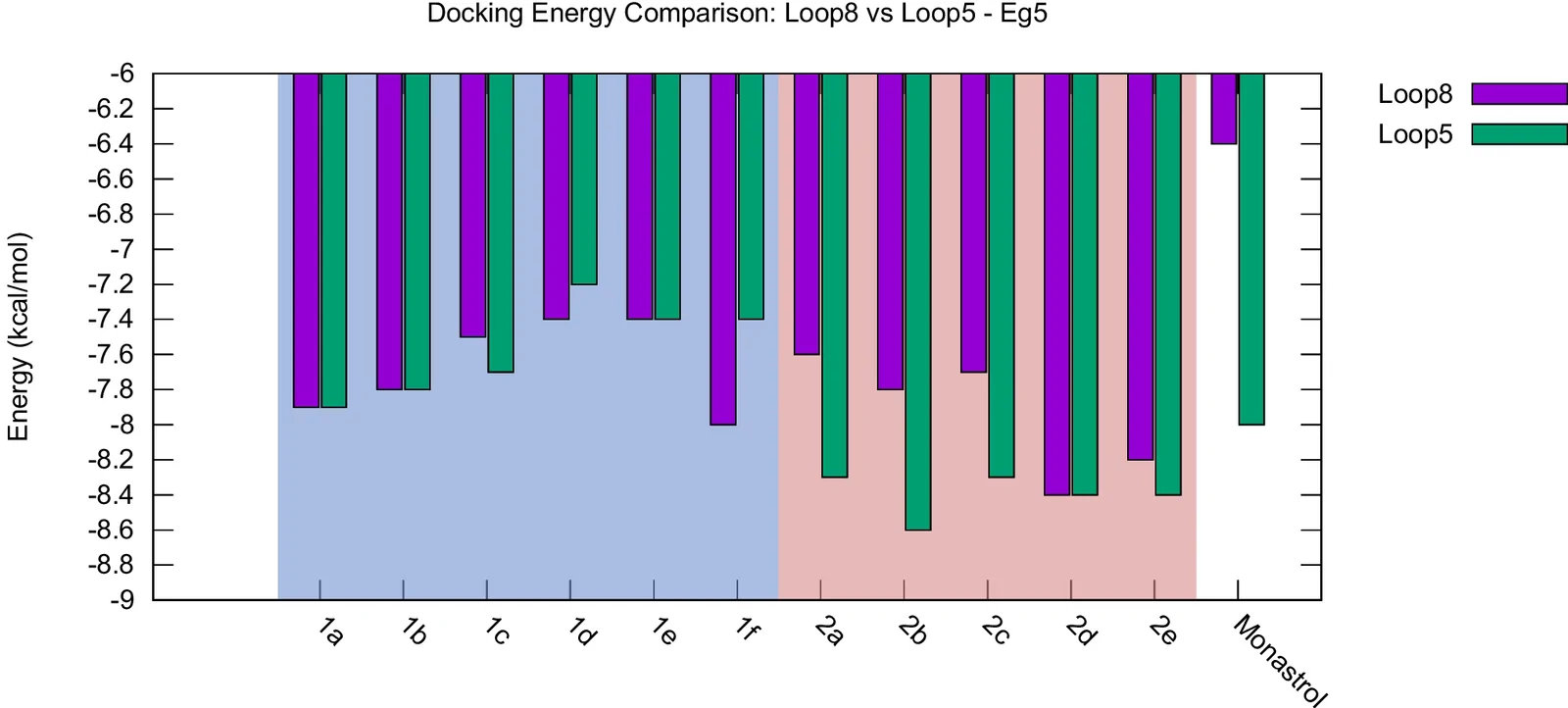
Grouped bar chart of the docking energies of **1a-f** (blue background), **2a-e** (red background), and monastrol at the loop5 (green bar) and loop8 (purple bar) sites

All derivatives showed favorable-energy poses at both the loop 5 site and the loop 8 site (Fig. 4). Considering the 11 compounds, the average energies were −7.79 kcal mol^−1^ at the loop 8 site and −7.95 kcal.mol^−1^ at the loop 5 site. The small difference between these means contrasts with the behavior of monastrol in the pockets, whose energy changed from −8.0 kcal.mol^−1^ at the loop 5 site to −6.4 kcal.mol^−1^ at the loop 8 site. Thus, whereas the reference ligand showed a preference of 1,6 kcal.mol^−1^ for the classical pocket, the thiazolopyrimidinethiones occupied both pockets with a small or nonexistent difference.

The series 1 compounds showed average values of −7.67 and −7.57 kcal.mol^−1^ for the loop 8 and loop 5 sites, respectively. Compounds **1a**, **1b**, and **1e** showed no difference between the sites, and compound **1f** favored the loop 8 site by 0.6 kcal.mol^−1^. Series 2 showed more favorable energies, especially at the loop 5 site, for which the average was −8.40 kcal.mol^−1^, compared with −7.94 kcal.mol^−1^ at the loop 8 site. Compound **2b** provided the best energy at the loop 5 site (−8.6 kcal.mol^−1^), whereas **2d** showed the best energy at the loop 8 site (−8.4 kcal.mol^−1^) and the same value at the loop 5 site. Compound **2e** also maintained high values at both sites (−8.2 and −8.4 kcal.mol^−1^). These results suggest that series 2 contains the best starting point for ligands with high apparent affinity, whereas series 1 offers useful examples for modulating preference between the pockets.

**Fig. 5:**
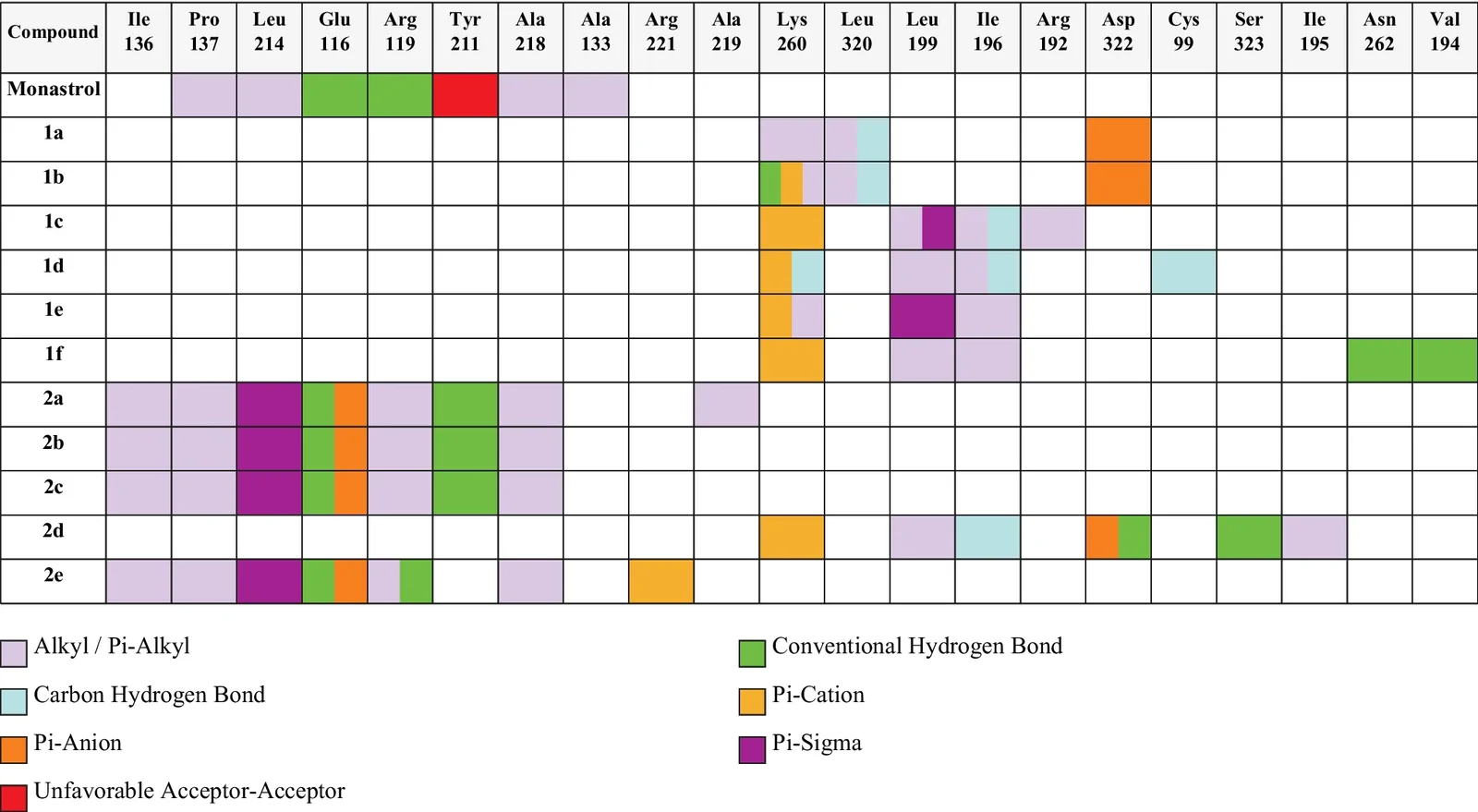
Interaction of the best pose of each studied compound with amino acids of the Eg5 motor domain (Ile136 to Ala219 = residues of the loop5 site; Lys260 to Val194 = residues of the loop8 site)

The loop 5 site is the allosteric pocket to which monastrol and several Eg5 inhibitors bind. In the evaluated poses, this environment involved residues such as Glu116, Gly117, Glu118, Arg119, Trp127, Asp130, Pro137, Tyr211, Leu214, Glu215, and Ala218. In contrast, the loop 8 site is located on the opposite face of the motor domain and corresponds to the S1 site previously identified by modeling and subsequently validated by direct binding, ATPase inhibition, and monopolar spindle formation. Its core includes hydrophobic residues such as Leu160, Leu161, Ile163, Ile196, Leu199, Val238, Val264, Ile319, and Leu320, as well as polar residues at the entrance, such as Ser159, Lys260, Asn262, Asp322, and Ser323. In this work, the designation “loop 8” was adopted because of the spatial proximity of the poses to segment 186–194 (Fig. 5).

**Fig. 6:**
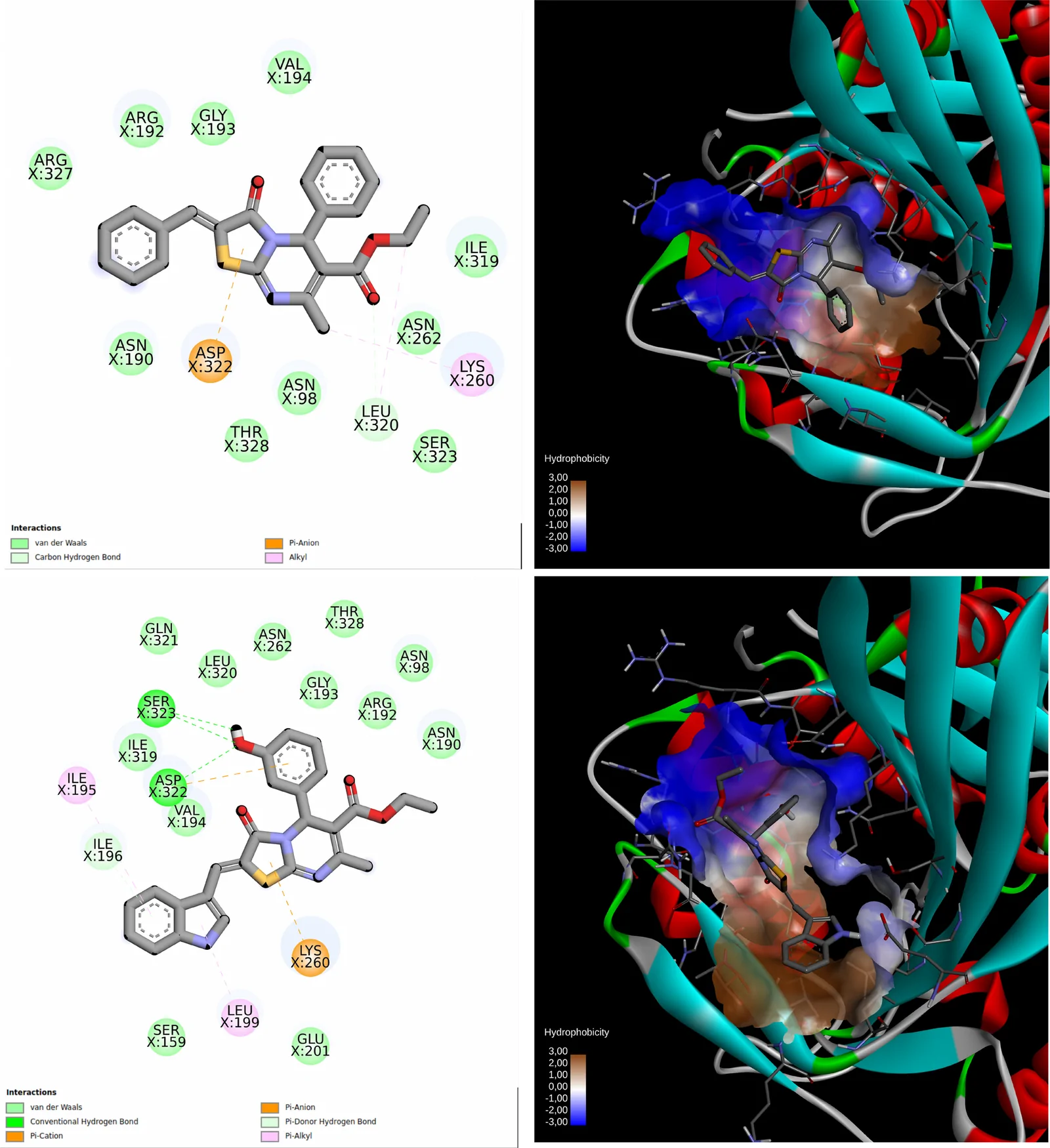
Two-dimensional diagrams and hydrophobicity map of the pocket for the representative poses of **1a** and **2d**, respectively

The poses of **1a** and **2d** illustrated the docking of the thiazolopyrimidinethiones to the second pocket. For **1a**, contacts were observed with Asn98, Asn190, Arg192, Gly193, Val194, Lys260, Asn262, Ile319, Leu320, Asp322, Ser323, Arg327, and Thr328, including a π-anion interaction with Asp322 and hydrophobic contact with Lys260 and Leu320. For **2d**, the predicted orientation approached Asp322 and Ser323, with hydrogen bonds, while the aromatic systems established π-cation or π-alkyl contacts with Lys260, Ile195, and Leu199. This pattern combines a predominantly hydrophobic core with polar anchoring points at the site entrance, similarly to that described for S1 ligands [5]. The corresponding docking poses are presented in Fig. 6.

The recurrence of poses compatible with two distinct allosteric pockets becomes more relevant than the small energy differences among the compounds. This property distinguishes them from monastrol-like compounds and may be advantageous in the face of changes in the classical site, since mutations in loop 5 are associated with resistance to inhibitors of this region [10, 32, 33]. However, the presence of a calculated pose at the loop 8 site alone does not demonstrate experimental occupation of this site or enzymatic inhibition. For this reason, the compound with the best docking result and the smallest energy difference between the sites in series 1 (**1a**) and the compound with the best docking result and the smallest energy difference between the sites in series 2 (**2d**) were evaluated by prolonged simulations at both sites.

### Molecular dynamics simulations

#### Global structural stability

**Fig. 7:**
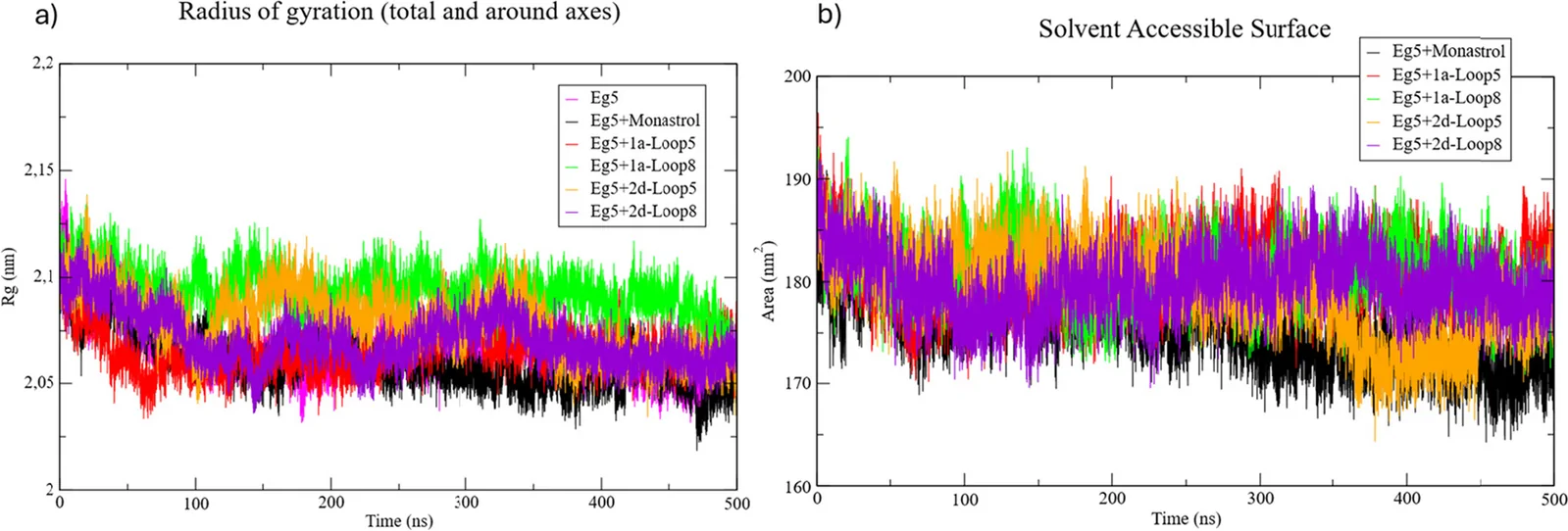
**a**) Radius of gyration of the Eg5-ADP motor domain and the complexes with monastrol, 1a, and 2 d at the loop 5 and loop 8 sites over 500 ns. **b**) Solvent-accessible surface area of the complexes with monastrol, 1a, and 2 d at the loop 5 and loop 8 sites over 500 ns

After molecular docking, the global compaction of the Eg5 motor domain was evaluated for the studied systems (Eg5-ADP, Eg5-ADP-monastrol, Eg5-ADP-**1a** at the loop 5 site, Eg5-ADP-**1a** at the loop 8 site, Eg5-ADP-**2d** at the loop 5 site, and Eg5-ADP-**2d** at the loop 8 site) through the radius of gyration (Rg). The Rg profiles remained without any progressive increase that would suggest loss of motor-domain folding over the 500 ns (Table 1 and Fig. 7). The Eg5-ADP system had an average Rg of 2.0628 ± 0.0091 nm. Values for the other systems ranged from 2.0568 ± 0.0098 nm for monastrol to 2,0921 ± 0.0094 nm for **1a** at the loop 8 site. Thus, even the largest difference relative to Eg5-ADP was only 0.0293 nm, supporting maintenance of the global structure, also observed in the RMSD profiles. The Rg of **1a** at loop 5 (2.0652 ± 0.0085 nm) was practically equal to the control, whereas the **2d**-loop 5 and **2d**-loop 8 systems showed 2.0763 ± 0.0140 and 2.0671 ± 0.0093 nm, respectively.

**Table 1:** Average radius of gyration and SASA for each system

System	Average Rg (nm)	Average SASA (nm^2^)
Eg5-ADP	2.0628 ± 0.0091	-
Monastrol-loop 5	2.0568 ± 0.0098	174.8076 ± 0.0245
1a-loop 5	2.0652 ± 0.0085	180.7902 ± 0.0191
1a-loop 8	2.0921 ± 0.0094	180.8779 ± 0.0213
2d-loop 5	2.0763 ± 0.0140	178.4699 ± 0.0293
2d-loop 8	2.0671 ± 0.0093	179.4799 ± 0.0197

Mean values correspond to the 100–500 ns interval, disregarding the initial period of the dynamics during which the systems are still stabilizing

The small increase in the Rg of **1a** at loop 8 relative to the other systems was not accompanied by a proportional increase in SASA. The average SASA values of **1a**-loop 5 and **1a**-loop 8 were very close, 180.7902 and 180.8779 nm^2^, respectively, although their radius of gyration differed by 0.0269 nm (Table 1). This change indicates that the compound did not have significant additional solvent exposure upon changing sites, but there was a small change in mass distribution relative to the center of the motor domain. For **2d**, occupation of loop 8 resulted in a slightly lower Rg than at loop 5, but a SASA approximately 1.01 nm^2^ higher (179.4799 versus 178.4699 nm^2^), indicating that binding at loop 8 for this compound alters packing but leaves the ligand more exposed to water.

Compared with monastrol, whose average SASA was 174.8076 nm^2^, the four complexes with the thiazolopyrimidinethiones showed higher average values, between 178.4699 and 180.8779 nm^2^ (Fig. 7). This difference is related to the larger surface area of the thiazolopyrimidine structures relative to monastrol and not to protein unfolding during the dynamics, since the Rg values are close.

### Stability of ligand binding and interaction energetics

The six systems showed RMSD profiles compatible with maintenance of motor-domain folding. The mean protein values ranged from 4.14 to 4.92 Å (Table 2). The **2d**-loop 5 complex showed the lowest mean protein RMSD (4.14 Å), whereas **1a**-loop 8 showed the highest value (4.92 Å). This difference was not accompanied by progressive loss of stability; the curves fluctuated predominantly on a plateau between approximately 4 and 5 Å, consistent with the presence of mobile loops in the Eg5 domain.

**Table 2:** Average RMSD and binding energy estimated by MM-PBSA

System	Protein RMSD (Å)	Ligand RMSD (Å)	MM-PBSA (kcal.mol^−1^)
Eg5-ADP	4.76	-	-
Monastrol-loop 5	4.65	1.24	−22.14 ± 0.07
1a-loop 5	4.40	1.12	−26.02 ± 0.07
1a-loop 8	4.92	1.00	−24.88 ± 0.09
2d-loop 5	4.14	1.53	−22.92 ± 0.12
2d-loop 8	4.51	1.23	−23.12 ± 0.13

**Fig. 8:**
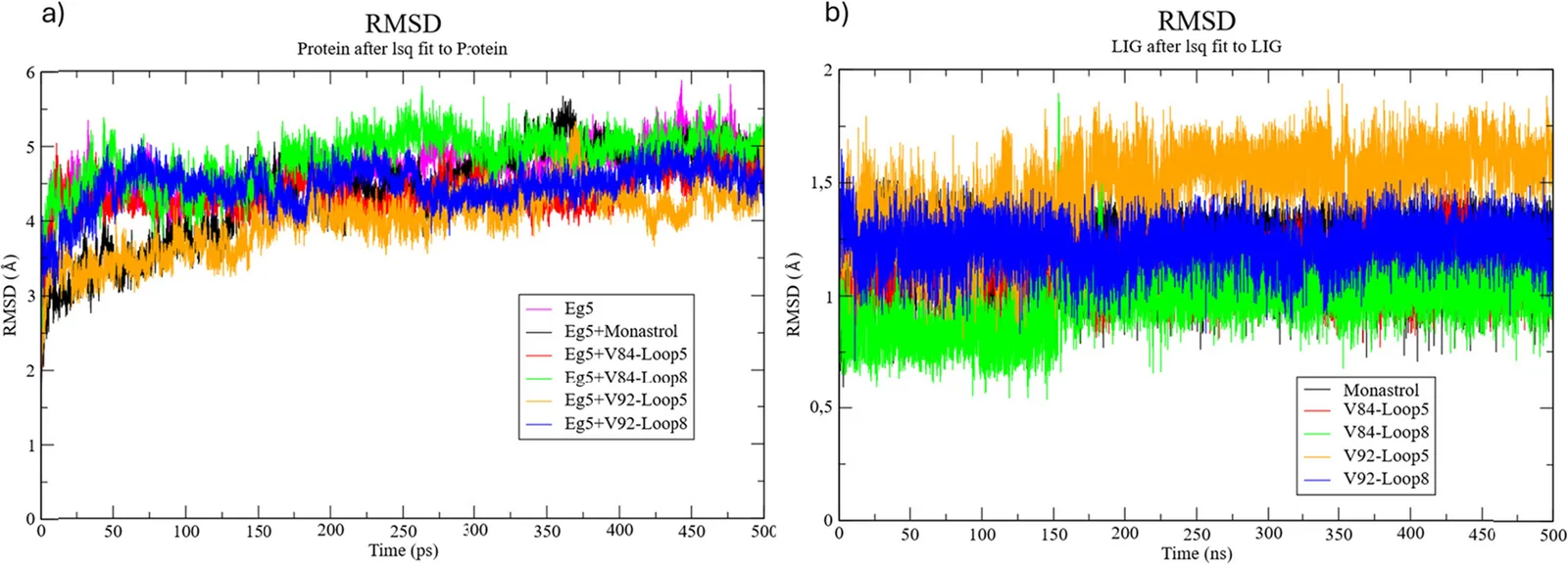
**a**) Protein RMSD for Eg5-ADP, monastrol, **1a**, and **2d** at both sites over 500 ns. **b**) Ligand RMSD after fitting to the ligand itself

The ligand RMSDs were substantially lower, ranging from 1.00 to 1.53 Å. The lowest value was obtained for **1a** at the loop 8 site (1.00 Å), followed by **1a** at loop 5 (1.12 Å), **2d** at loop 8 (1.23 Å), and monastrol (1.24 Å). The **2d**-loop 5 system showed the highest average (1.53 Å) and a plateau shift around 150 ns, but remained associated with the pocket throughout the remainder of the trajectory. Taken together, the RMSDs support the persistence of both orientations of **1a** and **2d** and justify comparison of their dynamic signatures (Fig. 8).

**Fig. 9:**
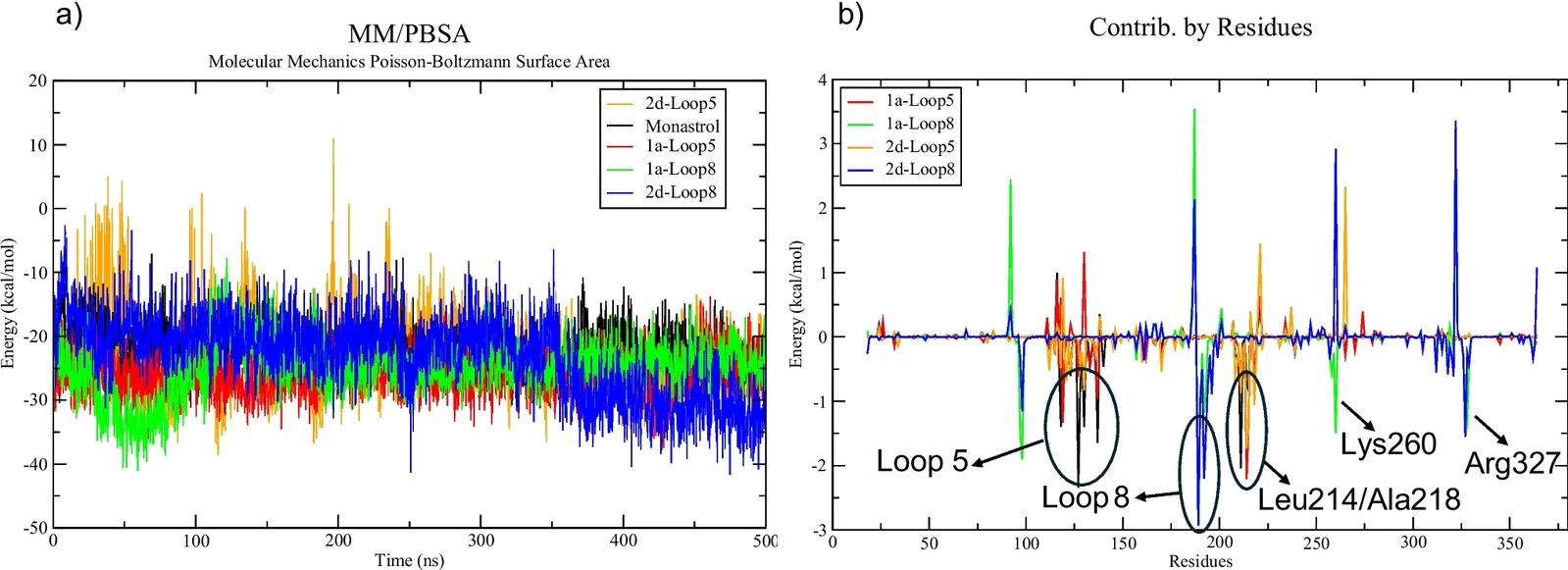
**a**) MM-PBSA energy as a function of time for monastrol and for **1a** and **2d** at both sites. **b**) Per-residue energy decomposition, highlighting residues 110–140 (loop5)/Leu214/Ala218 at the loop5 site and residues 189–194/Lys260/Arg327 at the loop8 site

The MM-PBSA estimates were negative for all complexes and maintained the same general order of magnitude as monastrol (Table 2 and Fig. 9). Compound **1a** showed the most favorable values, with −26.02 kcal.mol^−1^ at the loop 5 site and −24.88 kcal.mol^−1^ at the loop 8 site, compared with −22.14 kcal.mol^−1^ for monastrol. For **2d**, the estimates were −22.92 and −23.12 kcal.mol^−1^ at the loop 5 and loop 8 sites, respectively. The difference of only 0.20 kcal.mol^−1^ between the two poses of **2d** reinforces the dual-binding profile observed in docking. For **1a**, the difference was larger, but both values remained more favorable than the control.

Decomposition by residue clearly distinguished the two environments. At the loop 5 site, the strongest favorable contributions for **1a** were attributed to Leu214 (−2.21 kcal.mol^−1^), Glu215 (−1.36 kcal.mol^−1^), Arg119 (−1.34 kcal.mol^−1^), Pro137 (−0.96 kcal.mol^−1^), Tyr211 (−0.82 kcal.mol^−1^), and Ala218 (−0.74 kcal.mol^−1^). For **2d**, Leu214 (−1.74 kcal.mol^−1^), Glu215 (−1.40 kcal.mol^−1^), Ala218 (−1.06 kcal.mol^−1^), Tyr211 (−0.87 kcal.mol^−1^), and Asp130 (−0.83 kcal.mol^−1^) stood out. The recurrence of Leu214, Tyr211, and Ala218 is compatible with the role of these residues in delimiting the classical pocket [10, 11].

At the loop 8 site, the contributions were distributed between segment 189–194 (loop8) and residues forming the deep and lateral faces of the pocket. For **1a**, Arg189 (−2.91 kcal.mol^−1^), Asn98 (−1.90 kcal.mol^−1^), Lys260 (−1.50 kcal.mol^−1^), Thr328 (−1.35 kcal.mol^−1^), Arg327 (−1.19 kcal.mol^−1^), Arg192 (−1.18 kcal.mol^−1^), Asn190 (−1.13 kcal.mol^−1^), and Gly193 (−0.82 kcal.mol^−1^) stood out. For **2d**, the largest favorable contributions came from Arg189 (−2.93 kcal.mol^−1^), Arg192 (−2.20 kcal.mol^−1^), Arg327 (−1.56 kcal.mol^−1^), Gly193 (−1.44 kcal.mol^−1^), Asn98 (−1.16 kcal.mol^−1^), Asn190 (−0.99 kcal.mol^−1^), Ile196 (−0.89 kcal.mol^−1^), and Val194 (−0.71 kcal.mol^−1^).

When comparing the docking and dynamics results, residues Asp322 and Lys260, which participate in the interaction between compound **2d** and the loop8 site, show unfavorable mean contributions in MM-PBSA. On the other hand, loop8 residues (189–194) and Arg327 contributed to ligand stabilization during the trajectory. This indicates that system relaxation reorganized the residues that actually stabilize the ligand over time, indicating that the two-dimensional interaction maps obtained by docking for a single pose should not be treated as invariant.

Although docking and MM-PBSA energy results cannot be compared directly in numerical terms, there are nevertheless agreements between the results. Compounds **1a** and **2d** show favorable energies in both pockets and remain associated during the 500 ns simulation. Taken together, the data support the probability that the compounds bind to both allosteric sites of the Eg5 protein motor domain.

### Ligand-protein interaction network

Hydrogen-bond statistics and the persistence of the protein-ligand interaction network are summarized in Table 3. Compound **1a** formed comparatively few hydrogen bonds at either site, whereas 2 d maintained averages above one hydrogen bond at both pockets. The temporal contact patterns nevertheless showed that retention of each ligand was supported by a broader network of polar, hydrophobic, and van der Waals contacts rather than by hydrogen-bond counts alone.

**Table 3:** Descriptive summary of hydrogen bonds and persistent protein-ligand contacts during the 500 ns simulations

System; H bonds (mean ± SD)	Principal hydrogen bonds; occupancy (%)	Persistent/diagnostic contacts and temporal behavior
Monastrol-loop 5; 0.541 ± 0.603	Glu116, ligand donor (35.34); Arg119 (8.78); Glu116, protein donor (7.72)	Near-continuous Glu116, Gly117, Glu118, Arg119, Trp127, Ala133, Pro137, and Tyr211 contacts; Asp130 and Thr126 became evident later
**1a**-loop 5; 0.240 ± 0.428	Arg119, ligand donor (7.36); Arg221, protein donor (6.26); Leu214 (2.20); Tyr211 (2.15)	Broad and stable contact network throughout the trajectory, dominated by loop 5, α2, and α3 residues, with additional β4, β6, and Switch I contacts
**1a**-loop 8; 0.054 ± 0.243	Asn98 (16.89); Asn262 (14.19); Val194 (5.71)	Reorganization at approximately 150 ns; persistent Asn98/Asn190/Arg192/Gly193/Ile258/Lys260/Asp322/Thr328 axis maintained ligand retention
**2d**-loop 5; 1.191 ± 0.599	Glu116 (50.36); Glu215 (26.78); Glu118 (10.26)	Gradual transition between approximately 100 and 230 ns, followed by a stable contact geometry with recovery of loop 5/α2/α3 contacts and additional β-sheet/Switch I contacts
**2d**-loop 8; 1.286 ± 0.499	Asp322, principal acceptor; Lys260 (11.55); Arg327 (8.90)	Most uniform network, with persistent loop 8, β7, α5, and β8 contacts; Tyr97, Val238, and Ser240 became more continuous after approximately 350 ns

Hydrogen-bond values are mean ± standard deviation over 100–500 ns. Occupancies refer to the principal donor-acceptor pairs identified along the trajectories. Contact persistence is a descriptive classification derived from the 5 ns-binned temporal contact maps. Because each complex was represented by a single 500 ns trajectory, these descriptors support comparative interpretation but not inferential tests of statistical significance

**Fig. 10:**
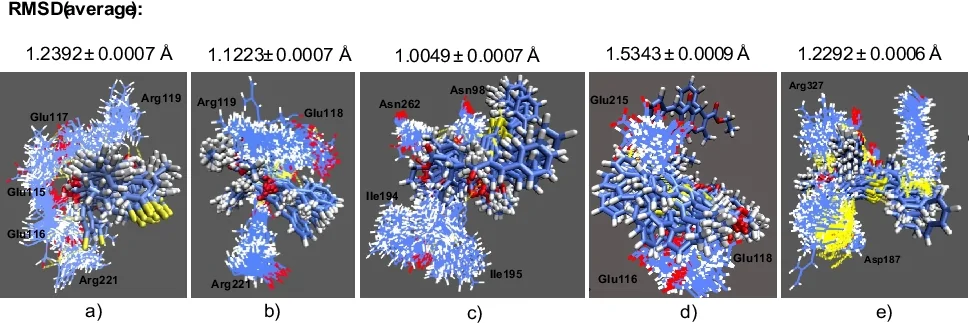
Frames of the compounds at the allosteric sites during 500 ns of simulation. **a**) Eg5 + Monastrol; **b**) Eg5 + **1a**-Loop5; **c**) Eg5 + **1a**-Loop8; **d**) Eg5 + **2d**-Loop5; **e**) Eg5 + **2d**-Loop8. Hydrogen bonds formed throughout the simulation are shown as yellow dashed lines. The RMSD average values show how much the different ligands fluctuated relative to themselves throughout the trajectory

The complete time-dependent hydrogen-bond traces and protein-ligand contact maps will be included in the Supplementary Information. Representative interaction frames are retained in Fig. 10.

### Dynamic effects on Eg5 allosteric communication

RMSF calculations revealed that binding of the compounds at the allosteric sites can redistribute the mobility (rigidifying or increasing the flexibility) of protein regions with communicating functions (Table 4). Compared with Eg5-ADP, all complexes increased the mean fluctuation of loop 5. Monastrol increased the RMSF of this region from 1.47 to 2.09 Å. The increases were more pronounced for **1a**-loop 5 (2.45 Å), **2d**-loop 5 (2.81 Å), and **2d**-loop 8 (2.48 Å). For both compounds, occupation of the loop 5 site itself produced the greatest local mobility, indicating that the presence of the ligand does not make the loop more rigid.

**Table 4:** Average RMSF of the functional regions of Eg5 (Å)

Region	Eg5-ADP	Monastrol	1a-loop 5	1a-loop 8	2d-loop 5	2d-loop 8
Cover-neck bundle, 25–40	4.03	3.63	3.99	3.09	3.63	2.54
Loop 5, 110–140	1.47	2.09	2.45	1.98	2.81	2.48
Loop 8, 186–194	2.60	1.54	1.80	2.08	2.39	2.02
Switch I, 226–236	1.61	2.16	2.93	2.70	2.78	2.59
Motor tip, 236–266	2.03	2.27	2.36	2.60	2.51	2.52
Switch II, 268–288	3.11	4.62	2.81	3.11	4.41	4.42
MT-binding site, 292–324	1.08	1.34	1.57	1.63	1.81	1.66
Neck linker, 356–364	1.69	2.12	2.16	2.15	2.37	2.16

The behavior of the loop 8 segment was the opposite: all ligands reduced its fluctuation relative to Eg5-ADP, whose average was 2.60 Å. Values ranged from 1.54 Å for monastrol to 2.39 Å for **2d**-loop 5. The reduction observed even when the ligand occupied the loop 5 site shows that the profile does not result only from direct contact; it reflects communication between distant regions of the motor domain. For the systems with the ligand at the loop 8 site, the average RMSFs were 2.08 Å for **1a** and 2.02 Å for **2d**, compatible with relative stabilization of segment 186–194 (loop8).

A particularly site-dependent effect was observed in the cover-neck bundle. Binding of **1a** at loop 5 practically did not change the average relative to Eg5-ADP (3.99 versus 4.03 Å), whereas the pose at loop 8 reduced it to 3.09 Å. For **2d**, the reduction was from 3.63 Å at loop 5 to 2.54 Å at loop 8. Therefore, occupation of the second pocket preferentially decreased the mobility of the N-terminal region involved in coupling with the neck linker. The neck linker itself showed the complementary effect: its mobility increased in all complexes, reaching 2.37 Å for **2d**-loop 5, compared with 1.69 Å in Eg5-ADP.

The Switch I, motor tip, and microtubule-binding interface regions also became more mobile in the presence of the compounds. The mean RMSF of Switch I increased from 1.61 Å in Eg5-ADP to 2.59–2.93 Å in the four complexes. At the microtubule-binding interface, the increase was from 1.08 Å to 1.57–1.81 Å, and was more pronounced for **2d**-loop 5. These segments form part of the network that connects nucleotide state to microtubule affinity and neck-linker orientation [34]. Thus, the coordinated increase in mobility in Switch I, the microtubule-binding surface, and the neck linker is consistent with perturbation of the transmission of chemical energy into mechanical energy by Eg5.

Switch II mobility made it possible to separate the effect of ligand structure from the effect of the site. For **1a**, the mean remained similar to or slightly lower than Eg5-ADP, with 2.81 Å at loop 5 and 3.11 Å at loop 8. For **2d**, however, the values increased to 4.41 and 4.42 Å, practically regardless of the occupied site and close to the monastrol profile (4.62 Å). Thus, the Switch II response appears to be determined mainly by the structure of **2d**, whereas the cover-neck bundle response was more sensitive to the location of the pose.

In summary, the RMSF results indicate that binding of the compounds does not produce stabilization caused by general rigidification of the Eg5 motor domain, but rather a redistribution of mobility, with some regions gaining mobility and others losing it. The increase in RMSF in loop5, Switch I, the microtubule-binding site, and the neck linker suggests that these regions are mechanically coupled. This behavior is significant because loop5 acts as a conformational lock capable of modulating changes in the following regions: the active site of the protein (ATP/ADP site), Switch I, the microtubule-binding site, and the neck linker [10]. Consistently, previous simulations showed that binding of allosteric inhibitors to loop 5 reorganizes communication pathways among the nucleotide, microtubule, and neck-linker sites, partially decoupling these functional regions [34]. The increased mobility of Switch II in systems with compound **2d** reinforces that series 2 compounds may impair ATP coupling and hydrolysis and, consequently, impair the conversion of chemical energy into mechanical energy more markedly than series 1 compounds.

The coordinated formation of the cover-neck bundle and positioning of the neck linker are important components of force generation by kinesins and of the Eg5 response to nucleotide state [35, 36]. Thus, the combination of lower cover-neck bundle mobility and higher neck-linker mobility may represent a loss of coordination and impairment of the protein step in the process of mitosis. The reduction in the RMSF of loop 8 even in systems with ligands at loop 5 also supports the occurrence of long-range allosteric communication. However, because RMSF reports only the amplitude of local fluctuations, without describing their direction or correlation, the hypothesis of decoupling between these regions should be tested by independent replicas and by analyses of correlated motions, principal components, or residue communication networks.

**Fig. 11:**
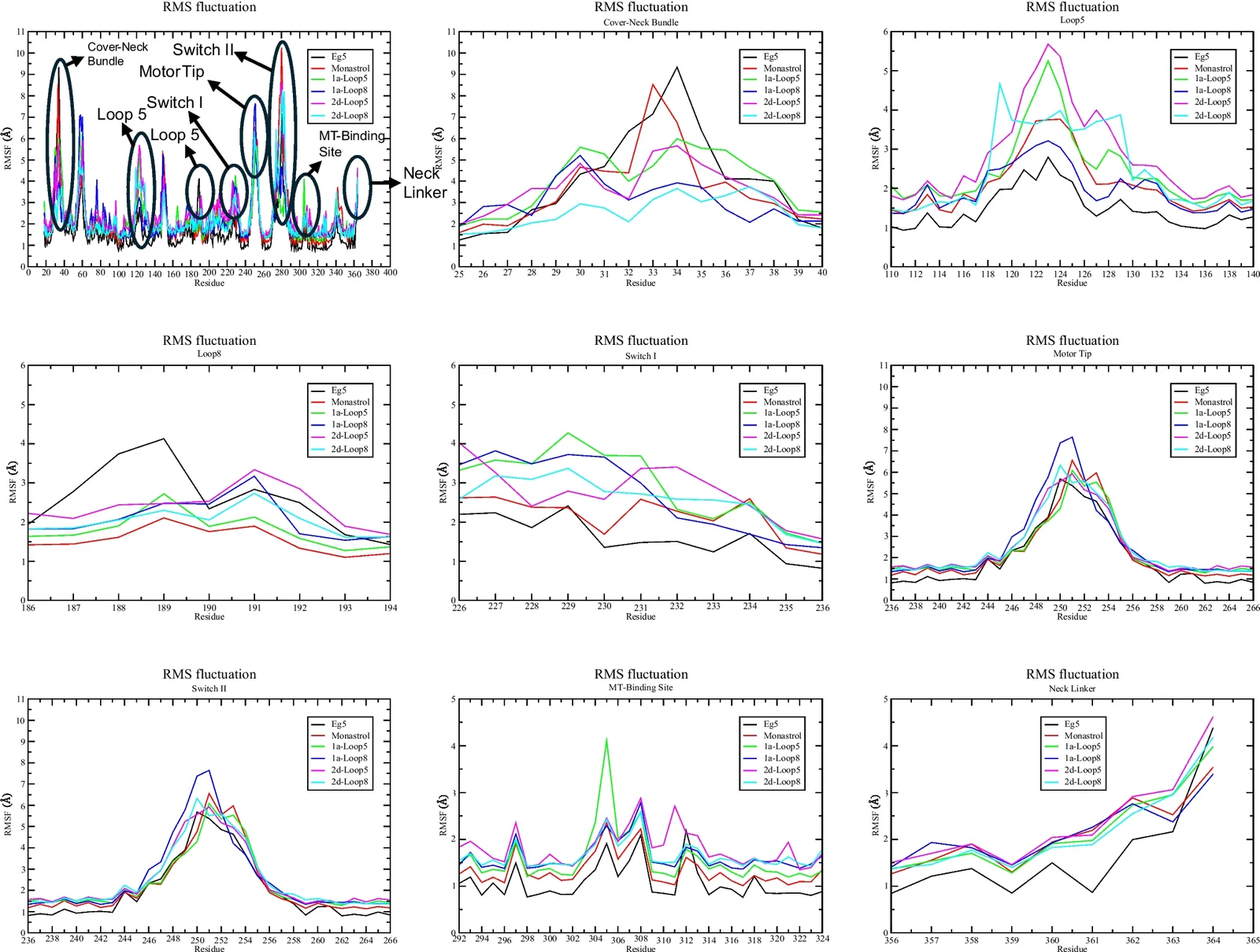
Overall per-residue RMSF profile for Eg5-ADP, monastrol, and the four complexes with **1a** and **2d**, accompanied by enlarged panels for the cover-neck bundle, loop 5, loop 8, Switch I, motor tip, Switch II, microtubule-binding site, and neck linker

**Fig. 12:**
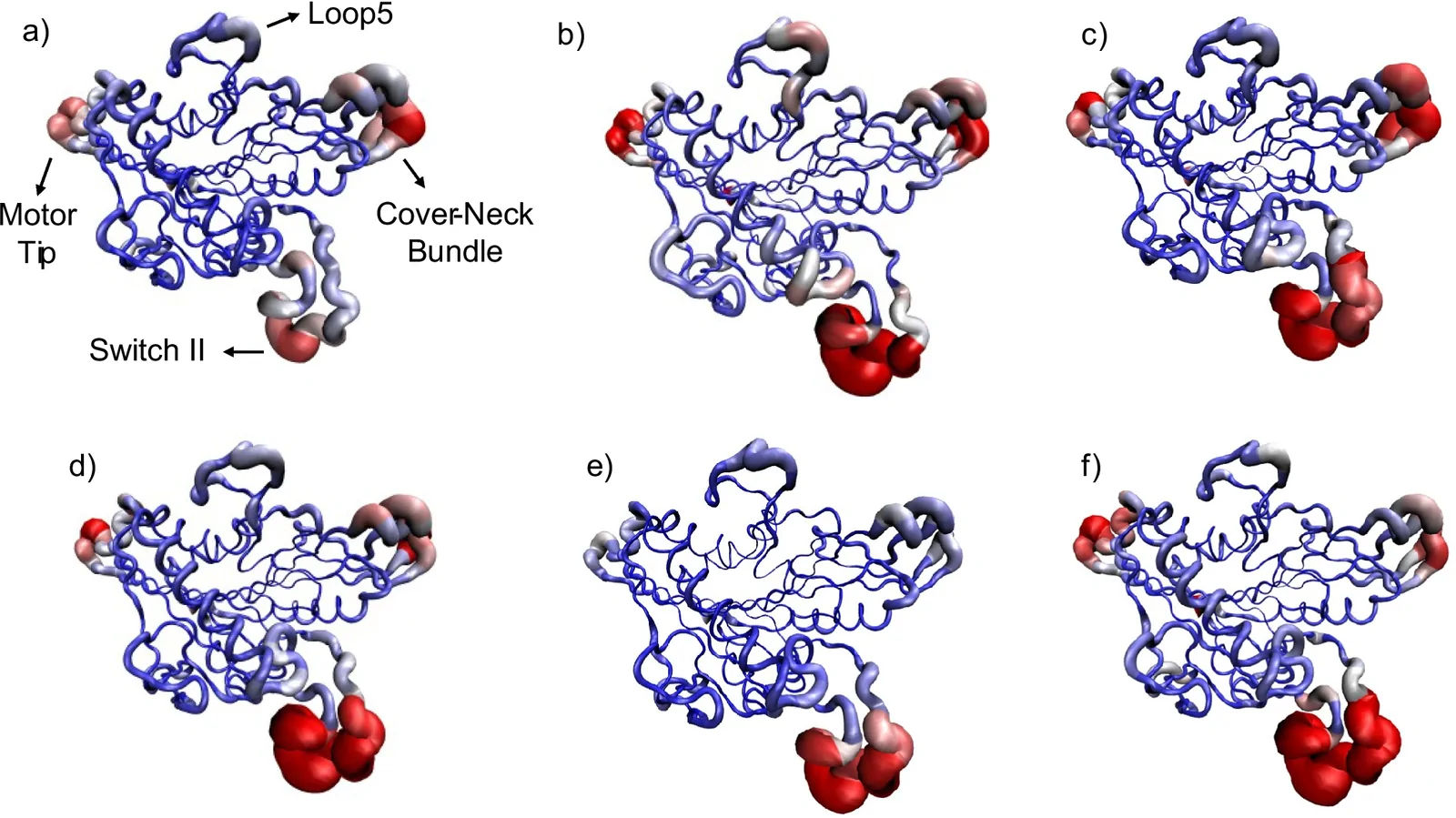
Spatial RMSF for the systems. **a**) Eg5 + ADP; **b**) Eg5 + Monastrol; **c**) Eg5 + **1a**-Loop5; **d**) Eg5 + **1a**-Loop8; **e**) Eg5 + **2d**-Loop5; **f**) Eg5 + **2d**-Loop8. The ligands were omitted from the figure for greater clarity

Figure 11 shows the overall RMSF profile in the systems, highlighting the cover-neck bundle, loop5, loop8, switch I, switch II, motor tip, MT-binding site, and neck linker structures. Figure 12 shows the spatial RMSF; larger red regions indicate greater flexibility, and smaller, thinner regions represent greater rigidity.

### Dynamic cross-correlation maps (DCCM)

The Dynamic Cross-Correlation Map (DCCM) analysis, based on 250 frames, was performed using the dccm_gui.py program developed by one of the authors [37]. Analysis of the Eg5 protein yielded a mean correlation close to zero (+ 0.0298), with 30% of the pairs exhibiting correlated motions and 24% exhibiting anticorrelated motions, indicating heterogeneous protein dynamics.

Among the ligands, **1a** showed greater internal coordination at loop 5 (+ 0.3861; 73% of the pairs were correlated) than at loop 8 (+ 0.1739; 56% correlated and 39% anticorrelated), where the larger displacement amplitude (RMSD = 1.8883 Å) suggests greater conformational heterogeneity. In contrast, **2d** exhibited predominantly concerted motions at both loop 5 (+ 0.6709; 90% correlated) and loop 8 (+ 0.6631; 97% correlated). At the former site, this behavior was accompanied by larger-amplitude displacements (RMSD = 2.9586 Å), whereas at loop 8, the combination of high correlation and lower displacement (RMSD = 0.8359 Å) indicates coordinated and more restricted internal dynamics. Monastrol also predominantly exhibited positive correlations (+ 0.5289; 90%). Thus, **2d**, particularly at loop 8, showed the greatest internal dynamic coherence, whereas **1a** at loop 8 displayed the most heterogeneous profile. The correlation map figures are provided in the Supplementary Information.

## Conclusion

The molecular modeling study revealed that thiazolopyrimidinethiones **1a-f** and **2a-e** are compatible with two allosteric sites of the Eg5 motor domain. Unlike monastrol, which showed a preference of 1.6 kcal.mol^−1^ for the classical loop 5 site, all derivatives were docked in both pockets with smaller differences. Compound **2b** showed the best score at loop 5, **2d** provided the best score at loop 8 and maintained the same energy at both sites, and **1f** showed the clearest preference for loop 8.

The 500 ns simulations showed that **1a** and **2d** remained associated with both sites, with ligand RMSDs between 1.00 and 1.53 Å and MM-PBSA energies comparable to or more favorable than that of monastrol. The per-residue energy decomposition results showed that Arg189, Asn98, Arg327, Arg192, Asn190, and Gly193 are the main residues for stabilization of the thiazolopyrimidines at the loop8 site. The stability of the radius of gyration and the behavior of SASA indicated preservation of global folding and predominantly local reorganizations. Compound **1a** formed few hydrogen bonds but maintained a broad contact network at loop 5 and reorganized its interface at loop 8. Compound **2d** showed more hydrogen bonds over time than the other compounds. Ligand retention therefore results from other intermolecular interactions and not from the simple number of hydrogen bonds. RMSF indicated a systematic redistribution of mobility: an increase in loop 5, Switch I, the microtubule-binding interface, and the neck linker, accompanied by a decrease in loop 8 and, especially for poses at the loop 8 site, in the cover-neck bundle. Compound **2d** also strongly increased the mobility of Switch II, regardless of the occupied site.

These findings support the hypothesis that binding at the loop 8 site may impair the conversion of chemical energy into mechanical energy by Eg5 through an allosteric route different from that initiated at loop 5, increasing the mobility of regions that coordinate the nucleotide, microtubule, and neck linker and consequently causing loss of coordination of the protein step followed by disruption of the mitotic spindle, potentially leading to apoptosis. The dual-binding ability identifies the thiazolopyrimidinethiones as a promising scaffold for exploring alternative allosteric sites of Eg5 and potentially circumventing limitations associated with the classical loop 5 pocket, such as mutations.

## Supplementary information

Below is the link to the electronic supplementary material.

Supplementary file1 (DOCX 61809 KB)

## Data Availability

The data supporting the findings of this study are available within the article and its Supplementary Information. The crystallographic structure of human kinesin Eg5 used in this study is publicly available in the Protein Data Bank under accession code 1X88. Additional datasets generated during the molecular docking and molecular dynamics analyses are available from the corresponding author upon reasonable request.
