# Supplementary material for: Molecular docking and molecular dynamics investigation of novels thiazolopyrimidines binding to canonical and alternative allosteric sites of kinesin Eg5
Source: J Mol Model. 2026 Sep 15;32(10):368. doi: 10.1007/s00894-026-06958-3 (PMC13578091; doi:10.1007/s00894-026-06958-3)
Supplement: Supplementary file 1 [file 894_2026_6958_MOESM1_ESM.docx]

**SUPPLEMENTARY INFORMATION**

**Molecular Docking and Molecular Dynamics Investigation of Thiazolopyrimidines Binding to Canonical and Alternative Allosteric Sites of Kinesin Eg5**

Vinícius S. Cunha¹, Arlan S. Gonçalves², Reginaldo B. dos Santos¹, Ricardo M. Kuster¹

¹ Department of Chemistry, Federal University of Espírito Santo, Av. Fernando Ferrari, Vitória 29075-910, Espírito Santo, Brazil

² Department of Chemistry, Federal Institute of Education, Science and Technology of Espírito Santo, Av. Min. Salgado Filho, Vila Velha 29106-010, Espírito Santo, Brazil

**Journal of Molecular Modeling**

**Contents**

- S1. Experimental information
- S2. Chemistry
- Table S1. Compound labels, substituents, and molecular formulae
- S3. Computational supporting details
- Tables S2-S4. Docking, molecular dynamics, and MM-PBSA parameters
- S4. Characterization spectra
- S5. Docking Studies
- S6. Molecular Dynamics Simulations

# S1. Experimental information

Commercial reagents and solvents were used without prior treatment. Reaction progress was monitored by thin-layer chromatography on silica gel UV254 plates (250 µm), with visualization under UV light and, when required, vanillin staining. Column chromatography was performed on silica gel 60 (70-230 mesh) with appropriate solvent mixtures.

Melting points are reported as observed. FTIR spectra were recorded on a PerkinElmer Spectrum 400 spectrometer using an ATR ZnSe accessory over 4000-400 cm⁻¹ (16 scans; 4 cm⁻¹ resolution). ¹H NMR (400 MHz) and ¹³C NMR (101 MHz) spectra were acquired on a Varian spectrometer in CDCl₃ or DMSO-d₆ with tetramethylsilane as internal standard. Chemical shifts (δ) are given in ppm and coupling constants (*J*) in Hz. Multiplicities are abbreviated as s, d, dd, t, td, q, qd, and m. Direct analyses were performed by Flow Injection Analysis coupled to Mass Spectrometry (FIA-MS) using a Vanquish Horizon UHPLC system coupled to an Orbitrap Exploris 480 mass spectrometer (Thermo Fisher Scientific, Waltham, MA, USA). The chromatographic column was removed and replaced with a zero-dead-volume union for direct sample introduction. Samples (3 µL injection volume) were introduced using an isocratic carrier stream of methanol at a flow rate of 0.20 mL min⁻¹ with a total acquisition time of 1.0 min per sample. Full-scan mass spectra were acquired over m/z 150–1000 at a resolving power of 120,000. MS/MS spectra were acquired in data-dependent acquisition (DDA) mode. Precursor ions were isolated using a 2.0 Da isolation window and fragmented by higher-energy collisional dissociation (HCD) with stepped normalized collision energies (NCE) of 15, 30, 70, and 100%. Dynamic exclusion was set to 10 s. Ionization was performed in positive ESI mode using the following source parameters: spray voltage, +3.5 kV; ion transfer tube temperature, 320 °C; vaporizer temperature (auxiliary gas heater), 275 °C; sheath gas, 35 (a.u.); auxiliary gas, 7 (a.u.); sweep gas, 1 (a.u.); and RF lens, 50%.

# S2. Chemistry

## S2.1. Preparation of ethyl chloroacetate

Chloroacetic acid (1.890 g, 20.0 mmol), ethanol (100 mL), and concentrated sulfuric acid (2.0 mL) were placed in a 250-mL round-bottom flask fitted with a magnetic stirrer and reflux condenser. The mixture was refluxed for 4 h while monitored by TLC, cooled to room temperature, and concentrated to approximately 25% of its initial volume. Ice-cold distilled water (50 mL) was added, and the mixture was transferred to a separatory funnel containing water (250 mL) and diethyl ether (100 mL). The organic layer was separated, neutralized with 10% aqueous sodium bicarbonate, dried over anhydrous magnesium sulfate, and concentrated under reduced pressure to give ethyl chloroacetate as a colorless oil (59%).

## S2.2. General preparation of DHPMT precursors

Choline chloride (0.140 g, 1.0 mmol) and thiourea (0.153 g, 2.0 mmol) were heated at 85 °C with magnetic stirring until a homogeneous deep eutectic solvent formed. Ethyl acetoacetate (1.0 mmol), the corresponding aromatic aldehyde (1.0 mmol), and two drops of concentrated hydrochloric acid were added. The mixture was stirred at 85 °C and monitored by TLC. After completion, ice-cold distilled water (approximately 10 mL) was added to precipitate the DHPMT precursor, which was collected by vacuum filtration and washed with ice-cold water. The corresponding precursors bearing phenyl, 4-chlorophenyl, 4-methoxyphenyl, 4-nitrophenyl, or 3-hydroxyphenyl substituents were used in the target-compound synthesis.

## S2.3. General synthesis of compounds 1a-f and 2a-e

Choline chloride (0.140 g, 1.0 mmol) and urea (0.120 g, 2.0 mmol) were heated at 85 °C until the deep eutectic solvent formed. The corresponding DHPMT (1.0 mmol), ethyl chloroacetate (160 µL, 1.5 mmol), and absolute ethanol (174 µL, 3.0 mmol) were added, and the mixture was stirred for 30 min. The corresponding aldehyde (1.0 mmol) was then added, and stirring at 85 °C was continued for 1 h while monitored by TLC. Absolute ethanol (6 mL) was added, and the reaction mixture was poured into ice-cold distilled water (150 mL). The precipitated solid was collected by vacuum filtration and dried at room temperature. When required, purification was achieved by washing the product with absolute ethanol (10 mL) for 30-40 min.

Table S1 Compound labels, substituents, and molecular formula

| **Compound** | **Series/substituents** | **Molecular formula** |
| --- | --- | --- |
| **1a** | R = H; R_1_ = H | C_23_H_20_N_2_O_3_S |
| **1b** | R = 4-Cl; R_1_ = H | C_23_H_19_ClN_2_O_3_S |
| **1c** | R = H; R_1_ = 4-OCH_3_ | C_24_H_22_N_2_O_4_S |
| **1d** | R = 4-OCH_3_; R_1_ = 4-OCH_3_ | C_25_H_24_N_2_O_5_S |
| **1e** | R = 4-OCH_3_; R_1_ = H | C_24_H_22_N_2_O_4_S |
| **1f** | R = 4-NO_2_; R_1_ = H | C_23_H_19_N_3_O_5_S |
| **2a** | R = H | C_25_H_21_N_3_O_3_S |
| **2b** | R = 4-Cl | C_25_H_20_ClN_3_O_3_S |
| **2c** | R = 4-OCH_3_ | C_26_H_23_N_3_O_4_S |
| **2d** | R = 3-OH | C_25_H_21_N_3_O_4_S |
| **2e** | R = 4-NO_2_ | C_25_H_20_N_4_O_5_S |

*For series 1, R denotes the substituent on the C5 aryl group and R_1_ denotes the substituent on the benzylidene ring. For series 2, R denotes the substituent on the C5 aryl group.*

## S2.4. Characterization data

### Ethyl (*Z*)-2-benzylidene-7-methyl-3-oxo-5-phenyl-2,3-dihydro-5*H*-thiazolo[3,2-*a*]pyrimidine-6-carboxylate (1a)

Yellow solid; yield 73%; m.p. 161-162 °C; molecular formula C_23_H_20_N_2_O_3_S.

**FTIR (ATR) νmax/cm⁻¹:** 3060, 1709, 1603, 1541, 1155. **¹H NMR** (400 MHz, CDCl_3_) δ: 7.74 (s, 1H), 7.48-7.44 (m, 4H), 7.43-7.40 (m, 3H), 7.33-7.26 (m, 3H), 6.20 (s, 1H), 4.12 (qd, *J* = 7.1, 2.8 Hz, 2H), 2.52 (s, 3H), 1.19 (t, *J* = 7.1 Hz, 3H). **¹³C NMR** (101 MHz, CDCl_3_) δ: 165.4, 165.2, 156.1, 152.3, 139.9, 133.4, 133.2, 130.4, 130.0, 129.2, 128.7, 128.6, 128.0, 120.2, 109.1, 60.5, 55.5, 22.7, 14.0. **HR-ESI-MS (positive mode):** *m/z* 405.1294 ([M+H]^+^, calc for C_23_H_21_N_2_O_3_S^+^ 405.1267). **MS/MS (HCD):** *m/z* 405.1257, 359.0842, 331.0895, 231.0219, 155.0485, 91.0540.

### Ethyl (*Z*)-2-benzylidene-5-(4-chlorophenyl)-7-methyl-3-oxo-2,3-dihydro-5*H*-thiazolo[3,2-*a*]pyrimidine-6-carboxylate (1b)

Yellow solid; yield 53%; m.p. 170-172 °C; molecular formula C_23_H_19_ClN_2_O_3_S

**FTIR (ATR) νmax/cm⁻¹:** 3056, 1707, 1606, 1562, 1230, 1157. **¹H NMR** (400 MHz, CDCl_3_) δ: 7.75 (s, 1H), 7.48-7.44 (m, 5H), 7.35 (d, *J* = 8.8 Hz, 2H), 7.28 (d, *J* = 8.8 Hz, 2H), 6.17 (s, 1H), 4.12 (qd, *J* = 7.1, 2.8 Hz, 2H), 2.52 (s, 3H), 1.20 (t, *J* = 7.1 Hz, 3H). **¹³C NMR** (101 MHz, CDCl_3_) δ: 165.3, 165.2, 156.2, 152.7, 138.4, 134.6, 133.7, 133.1, 130.6, 130.1, 129.5, 129.3, 128.8, 119.9, 108.6, 60.6, 54.8, 22.7, 14.1. **HR-ESI-MS (positive mode):** *m/z* 439.0866 ([M+H]^+^, calc for C_23_H_20_ClN_2_O_3_S^+^ 439.0878). **MS/MS (HCD):** *m/z* 439.0866, 393.0452, 231.0221, 189.0078, 91.0541.

### Ethyl (*Z*)-2-(4-methoxybenzylidene)-7-methyl-3-oxo-5-phenyl-2,3-dihydro-5*H*-thiazolo[3,2-*a*]pyrimidine-6-carboxylate (1c)

Yellow solid; yield 50%; m.p. 178-180 °C; molecular formula C_24_H_22_N_2_O_4_S.

**FTIR (ATR) νmax/cm⁻¹:** 3004, 1702, 1616, 1543, 1154. **¹H NMR** (400 MHz, CDCl_3_) δ: 7.69 (s, 1H), 7.43-7.40 (m, 4H), 7.31-7.25 (m, 3H), 6.97 (d, *J* = 8.8 Hz, 2H), 6.19 (s, 1H), 4.12 (qd, *J* = 7.1, 2.8 Hz, 2H), 3.85 (s, 3H), 2.52 (s, 3H), 1.19 (t, *J* = 7.1 Hz, 3H). **¹³C NMR** (101 MHz, CDCl_3_) δ: 165.6, 165.5, 161.4, 156.6, 152.6, 140.2, 133.4, 132.1, 128.6, 128.5, 128.0, 125.9, 117.2, 114.8, 108.9, 60.4, 55.5, 55.4, 22.8, 14.1. **HR-ESI-MS (positive mode):** *m/z* 435.1367 ([M+H]^+^, calc for C_24_H_23_N_2_O_4_S^+^ 435.1373). **MS/MS (HCD):** *m/z* 435.1364, 389.0954, 261.0324, 155.0489, 121.0646.

### Ethyl (*Z*)-2-(4-methoxybenzylidene)-5-(4-methoxyphenyl)-7-methyl-3-oxo-2,3-dihydro-5*H*-thiazolo[3,2-*a*]pyrimidine-6-carboxylate (1d)

Yellow solid; yield 81%; m.p. 145-146 °C; molecular formula C_25_H_24_N_2_O_5_S.

**FTIR (ATR) νmax/cm⁻¹:** 2974, 1700, 1588, 1508, 1252, 1228. **¹H NMR** (400 MHz, CDCl_3_) δ: 7.69 (s, 1H), 7.42 (d, *J* = 8.8 Hz, 2H), 7.33 (d, *J* = 8.8 Hz, 2H), 6.97 (d, *J* = 8.8 Hz, 2H), 6.81 (d, *J* = 8.8 Hz, 2H), 6.15 (s, 1H), 4.12 (qd, *J* = 7.1, 2.8 Hz, 2H), 3.85 (s, 3H), 3.76 (s, 3H), 2.51 (s, 3H), 1.19 (t, *J* = 7.1 Hz, 3H). **¹³C NMR** (101 MHz, CDCl_3_) δ: 165.6, 165.5, 161.4, 159.6, 156.4, 152.2, 133.3, 132.5, 132.0, 129.3, 125.9, 117.3, 114.8, 113.9, 108.9, 60.4, 55.5, 55.2, 54.8, 22.7, 14.1. **HR-ESI-MS (positive mode):** *m/z* 465.1472 ([M+H]^+^, calc for C_25_H_25_N_2_O_5_S^+^ 465.1479). **MS/MS (HCD):** *m/z* 465.1468, 419.1052, 391.1108, 261.0326, 185.0588.

### Ethyl (*Z*)-2-benzylidene-5-(4-methoxyphenyl)-7-methyl-3-oxo-2,3-dihydro-5*H*-thiazolo[3,2-*a*]pyrimidine-6-carboxylate (1e)

Yellow solid; yield 65%; m.p. 145-146 °C; molecular formula C_24_H_22_N_2_O_4_S.

**FTIR (ATR) νmax/cm⁻¹:** 2976, 1707, 1606, 1541, 1228, 1157. **¹H NMR** (400 MHz, CDCl_3_) δ: 7.74 (s, 1H), 7.48-7.41 (m, 5H), 7.34 (d, *J* = 8.8 Hz, 2H), 6.82 (d, *J* = 8.8 Hz, 2H), 6.16 (s, 1H), 4.12 (qd, *J* = 7.1, 2.8 Hz, 2H), 3.76 (s, 3H), 2.52 (s, 3H), 1.19 (t, *J* = 7.1 Hz, 3H). **¹³C NMR** (101 MHz, CDCl_3_) δ: 165.5, 165.3, 159.7, 155.9, 152.0, 133.3, 133.2, 132.2, 130.4, 130.0, 129.4, 129.2, 120.4, 113.9, 109.3, 60.4, 55.2, 54.9, 22.7, 14.1. **HR-ESI-MS (positive mode):** *m/z* 435.1368 ([M+H]^+^, calc for C_24_H_23_N_2_O_4_S^+^ 435.1373). **MS/MS (HCD):** *m/z* 435.1369, 389.0957, 261.0326, 155.0489.

### Ethyl (*Z*)-2-benzylidene-7-methyl-5-(4-nitrophenyl)-3-oxo-2,3-dihydro-5*H*-thiazolo[3,2-*a*]pyrimidine-6-carboxylate (1f)

Yellow solid; yield 30%; m.p. 138-140 °C; molecular formula C_23_H_19_N_3_O_5_S.

**FTIR (ATR) νmax/cm⁻¹:** 2976, 1710, 1684, 1522, 1231, 1159. **¹H NMR** (400 MHz, CDCl_3_) δ: 8.18 (d, *J* = 8.8 Hz, 2H), 7.75 (s, 1H), 7.60 (d, *J* = 8.8 Hz, 2H), 7.48-7.44 (m, 5H), 6.24 (s, 1H), 4.12 (qd, *J* = 7.1, 2.8 Hz, 2H), 2.54 (s, 3H), 1.20 (t, *J* = 7.1 Hz, 3H). **¹³C NMR** (101 MHz, CDCl_3_) δ: 170.2, 165.0, 156.4, 153.5, 150.3, 147.9, 146.5, 134.4, 130.8, 130.1, 129.3, 129.1, 123.9, 119.5, 107.9, 60.8, 54.8, 22.9, 14.1. **HR-ESI-MS (positive mode):** *m/z* 450.1116 ([M+H]^+^, calc for C_23_H_20_N_3_O_5_S^+^ 450.1118). **MS/MS (HCD):** *m/z* 450.1109, 404.0692, 376.0760, 231.0220, 200.0333, 91.0542.

### Ethyl (*Z*)-2-[(1H-indol-3-yl)methylene]-7-methyl-3-oxo-5-phenyl-2,3-dihydro-5*H*-thiazolo[3,2-*a*]pyrimidine-6-carboxylate (2a)

Yellow solid; yield 53%; m.p. 280-282 °C; molecular formula C_25_H_21_N_3_O_3_S.

**FTIR (ATR) νmax/cm⁻¹:** 3325, 1703, 1603, 1539, 1165, 1079. **¹H NMR** (400 MHz, DMSO-d_6_) δ: 8.05 (s, 1H), 7.88-7.84 (m, 2H), 7.50 (d, *J* = 8.0 Hz, 1H), 7.37-7.28 (m, 5H), 7.25 (td, *J* = 6.8, 1.2 Hz, 1H), 7.18 (td, *J* = 6.8, 0.8 Hz, 1H), 6.06 (s, 1H), 4.06 (qd, *J* = 7.1, 2.8 Hz, 2H), 2.39 (s, 3H), 1.13 (t, *J* = 7.1 Hz, 3H). **¹³C NMR** (101 MHz, DMSO-d_6_) δ: 165.5, 164.8, 156.6, 152.3, 141.3, 136.7, 129.6, 129.1, 128.9, 127.8, 127.2, 126.2, 123.7, 121.7, 118.9, 112.9, 112.6, 110.9, 108.4, 60.5, 55.2, 22.9, 14.3. **HR-ESI-MS (positive mode):** *m/z* 444.1374 ([M+H]^+^, calc for C_25_H_22_N_3_O_3_S^+^ 444.1376). **MS/MS (HCD):** *m/z* 444.1369, 398.0961, 270.0329, 155.0488.

### Ethyl (*Z*)-5-(4-chlorophenyl)-2-[(1*H*-indol-3-yl)methylene]-7-methyl-3-oxo-2,3-dihydro-5*H*-thiazolo[3,2-*a*]pyrimidine-6-carboxylate (2b)

Yellow solid; yield 40%; m.p. 300dec °C; molecular formula C_25_H_20_ClN_3_O_3_S.

**FTIR (ATR) νmax/cm⁻¹:** 3176, 1707, 1699, 1524, 1223, 1068. **¹H NMR** (400 MHz, DMSO-d_6_) δ: 12.23 (s, 1H, NH), 8.05 (s, 1H), 7.88-7.84 (m, 2H), 7.50 (d, *J* = 8.0 Hz, 1H), 7.42 (d, *J* = 8.0 Hz, 2H), 7.34 (d, *J* = 8.0 Hz, 2H), 7.24 (td, *J* = 7.2, 0.8 Hz, 1H), 7.18 (td, *J* = 7.2, 0.8 Hz, 1H), 6.04 (s, 1H), 4.06 (qd, *J* = 7.1, 2.8 Hz, 2H), 2.39 (s, 3H), 1.13 (t, *J* = 7.1 Hz, 3H). **¹³C NMR** (101 MHz, DMSO-d_6_) δ: 165.3, 164.7, 156.6, 152.7, 140.1, 136.7, 133.4, 129.8, 129.7, 129.1, 127.2, 126.4, 123.6, 121.7, 118.9, 112.9, 112.4, 110.9, 107.9, 60.5, 54.6, 23.0, 14.3. **HR-ESI-MS (positive mode):** *m/z* 478.0983 ([M+H]^+^, calc for C_25_H_21_ClN_3_O_3_S^+^ 478.0987). **MS/MS (HCD):** *m/z* 478.0978, 432.0561, 404.0615, 270.0331, 189.0075, 130.0651.

### Ethyl (*Z*)-2-[(1*H*-indol-3-yl)methylene]-5-(4-methoxyphenyl)-7-methyl-3-oxo-2,3-dihydro-5*H*-thiazolo[3,2-*a*]pyrimidine-6-carboxylate (2c)

Yellow solid; yield 56%; m.p. 275-276 °C; molecular formula C_26_H_23_N_3_O_4_S.

**FTIR (ATR) νmax/cm⁻¹:** 3181, 1705, 1601, 1532, 1224, 1163. **¹H NMR** (400 MHz, DMSO-d_6_) δ: 12.20 (d, *J* = 3.1 Hz, 1H, NH), 8.04 (s, 1H), 7.86 (d, *J* = 7.9 Hz, 1H), 7.83 (d, *J* = 3.0 Hz, 1H), 7.50 (dd, *J* = 8.0, 1.0 Hz, 1H), 7.26-7.16 (m, 4H), 6.89 (d, *J* = 8.8 Hz, 2H), 6.00 (s, 1H), 4.06 (qd, *J* = 7.1, 2.8 Hz, 2H), 3.70 (s, 3H), 2.39 (s, 3H), 1.13 (t, *J* = 7.1 Hz, 3H). **¹³C NMR** (101 MHz, DMSO-d_6_) δ: 165.5, 164.8, 159.6, 156.3, 152.1, 136.7, 133.4, 129.5, 129.1, 127.2, 126.1, 123.6, 121.6, 118.9, 114.4, 112.9, 112.7, 110.9, 108.4, 60.5, 55.5, 54.6, 22.9, 14.3. **HR-ESI-MS (positive mode)**: *m/z* 474.1477 ([M+H]^+^, calc for C_26_H_24_N_3_O_4_S^+^ 474.1482). **MS/MS (HCD):** *m/z* 474.1476, 428.1060, 400.1089, 270.0331, 185.0590, 130.0650.

### Ethyl (*Z*)-5-(3-hydroxyphenyl)-2-[(1*H*-indol-3-yl)methylene]-7-methyl-3-oxo-2,3-dihydro-5*H*-thiazolo[3,2-*a*]pyrimidine-6-carboxylate (2d)

Yellow solid; yield 36%; m.p. 300dec °C; molecular formula C_25_H_21_N_3_O_4_S.

**FTIR (ATR) νmax/cm⁻¹:** 3353, 1707, 1685, 1541, 1223, 1148. **¹H NMR** (400 MHz, DMSO-d_6_) δ: 12.23 (d, *J* = 3.1 Hz, 1H, NH), 9.50 (s, 1H, OH), 8.06 (s, 1H), 7.87-7.83 (m, 2H), 7.50 (dd, *J* = 8.0, 1.0 Hz, 1H), 7.24 (td, *J* = 7.2, 1.2 Hz, 1H), 7.18 (td, *J* = 8.0, 1.2 Hz, 1H), 7.12 (t, *J* = 8.1 Hz, 1H), 6.72 (m, 2H), 6.66 (dd, *J* = 8.1, 1.1 Hz, 1H), 5.96 (s, 1H), 4.08 (qd, *J* = 7.1, 2.8 Hz, 2H), 2.37 (s, 3H), 1.16 (t, *J* = 7.1 Hz, 3H). **¹³C NMR** (101 MHz, DMSO-d_6_) δ: 165.5, 164.7, 157.9, 156.6, 152.1, 142.5, 136.7, 130.1, 129.5, 127.2, 126.2, 121.6, 118.8, 118.2, 115.8, 114.4, 112.9, 112.6, 110.9, 108.5, 60.5, 54.9, 22.9, 14.4. **HR-ESI-MS (positive mode):** *m/z* 460.1323 ([M+H]^+^, calc for C_25_H_22_N_3_O_4_S^+^ 460.1326). **MS/MS (HCD):** *m/z* 460.1316, 414.0902, 244.0537, 171.0435, 130.0651.

### Ethyl (*Z*)-2-[(1H-indol-3-yl)methylene]-7-methyl-5-(4-nitrophenyl)-3-oxo-2,3-dihydro-5*H*-thiazolo[3,2-*a*]pyrimidine-6-carboxylate (2e)

Yellow solid; yield 40%; m.p. 295-297 °C; molecular formula C_25_H_20_N_4_O_5_S.

**FTIR (ATR) νmax/cm⁻¹:** 3171, 1707, 1673, 1517, 1226, 1151. **¹H NMR** (400 MHz, CDCl_3_) δ: 8.96 (d, *J* = 3.1 Hz, 1H, NH), 8.19 (d, *J* = 8.0 Hz, 2H), 8.13 (s, 1H), 7.78 (dd, *J* = 8.0, 1.2 Hz, 1H), 7.63 (d, *J* = 8.0 Hz, 2H), 7.56 (d, *J* = 3.1, 0.7 Hz, 1H), 7.44 (d, *J* = 7.6 Hz, 1H), 7.32 (td, *J* = 8.2, 1.3 Hz, 1H), 7.28 (td, *J* = 7.9, 1.1 Hz, 1H), 6.30 (s, 1H), 4.13 (q, *J* = 7.1 Hz, 2H), 2.56 (s, 3H), 1.21 (t, *J* = 7.1 Hz, 3H). **¹³C NMR** (101 MHz, CDCl_3_) δ: 165.2, 165.0, 156.7, 153.8, 147.8, 146.9, 135.8, 129.1, 126.9, 126.4, 124.2, 123.9, 122.1, 118.7, 113.7, 112.4, 111.8, 107.4, 60.7, 54.7, 23.4, 14.1. **HR-ESI-MS (positive mode):** *m/z* 489.1222 ([M+H]^+^, calc for C_25_H_21_N_4_O_5_S^+^ 489.1227). **MS/MS (HCD):** *m/z* 489.1215, 443.0795, 270.0330, 200.0338, 130.0651.

# S3. Computational supporting details

Table S2 Molecular docking and redocking parameters

| **Parameter** | **Setting** |
| --- | --- |
| Protein structure | Human Eg5 motor domain, PDB 1X88; chain A retained with Mg²⁺ and ADP |
| Modeled segment | Switch II residues Ile272-Gly286 reconstructed with SWISS-MODEL |
| Protein protonation/preparation | pH 7.4 with PDB2PQR; AMBER parameters; polar H atoms and Kollman charges assigned in AutoDock Tools 1.5.7 |
| Ligand preparation | Structures generated for pH 7.4 and geometry-optimized with Open Babel |
| Docking engine | QuickVina-W |
| Grid box | 46 × 40 × 40 Å; center x = 31.073, y = 31.557, z = 103.733 |
| Exhaustiveness | 32 |
| Sampling | 20 independent runs per ligand; up to 20 modes per run |
| Pose selection | Lowest-energy pose in the loop 5 site and lowest-energy pose in the loop 8/S1 site |
| Redocking validation | Monastrol RMSD = 1.10 Å; docking score = -8.0 kcal mol⁻¹ |

Table S3 Molecular dynamics simulation parameters

| **Parameter** | **Setting** |
| --- | --- |
| Software | GROMACS |
| Systems | Eg5-ADP; Eg5-ADP-monastrol; Eg5-ADP-1a and Eg5-ADP-2d in the loop 5 and loop 8 sites |
| Force field / water | OPLS-AA/L / TIP3P |
| Ligand parameters | MMFF94 pre-optimization followed by ACPYPE parameterization adapted to OPLS-AA/L |
| Periodic box | Dodecahedral; minimum solute-to-box distance 1.5 nm |
| Minimization | Steepest descent with positional restraints; steepest descent without restraints; L-BFGS |
| Equilibration | 500 ps with positional restraints |
| Production | 500 ns without positional restraints |
| Integration time step | 2 fs |
| Temperature coupling | 310 K; velocity-rescale thermostat; τT = 0.1 ps |
| Pressure coupling | 1 bar; isotropic Berendsen barostat; τP = 1 ps; compressibility = 4.5 × 10⁻⁵ bar⁻¹ |
| Electrostatics / van der Waals | Particle mesh Ewald electrostatics; 1.0-nm Coulomb and van der Waals cutoffs |
| Bond constraints | All bonds constrained with LINCS; order 4 |
| Trajectory output | Coordinates every 20 ps |
| RMSD averaging | First 100 ns excluded |

Table S4 MM-PBSA calculation parameters

| **Parameter** | **Setting** |
| --- | --- |
| Software | g_mmpbsa |
| Polar solvation | Linearized Poisson-Boltzmann equation |
| Solute / solvent dielectric constants | 2 / 80 |
| Ionic strength | 0.150 mol L⁻¹ for monovalent positive and negative ions |
| Temperature | 310 K |
| Solvent probe radius | 1.4 Å |
| Charge mapping / dielectric surface | Cubic B-spline (spl4) / smoothed molecular surface |
| Apolar contribution | SASA model; γ = 0.0226778 kJ mol⁻¹ Å⁻²; probe radius = 1.4 Å |
| Reported quantities | Mean binding energy and per-residue energy decomposition |

## S3.1. Additional validation and dynamic analyses

Fig. S1 Ramachandran plot for the amino acids of the final Eg5 model.

Table S5 Docking energies of the thiazolopyrimidinethiones at the two allosteric sites of Eg5

| **Compound** | **Loop 8 site (kcal.mol-1)** | **Loop 5 site (kcal.mol-1)** | **ΔE (loop 8 - loop 5)** |
| --- | --- | --- | --- |
| **1a** | -7.9 | -7.9 | 0.0 |
| **1b** | -7.8 | -7.8 | 0.0 |
| **1c** | -7.5 | -7.7 | +0.2 |
| **1d** | -7.4 | -7.2 | -0.2 |
| **1e** | -7.4 | -7.4 | 0.0 |
| **1f** | -8.0 | -7.4 | -0.6 |
| **2a** | -7.6 | -8.3 | +0.7 |
| **2b** | -7.8 | -8.6 | +0.8 |
| **2c** | -7.7 | -8.3 | +0.6 |
| **2d** | -8.4 | -8.4 | 0.0 |
| **2e** | -8.2 | -8.4 | +0.2 |
| **Monastrol** | -6.4 | -8.0 | +1.6 |

**Note:** ΔE values < 0 indicate favoring of the loop 8 site, whereas ΔE values > 0 indicate favoring of the loop 5 site.

Fig. S2 Number of hydrogen bonds between the protein and ligand over the 500 ns trajectories for monastrol, 1a-loop 5, 1a-loop 8, 2d-loop 5, and 2d-loop 8.

Fig. S3 Temporal protein-ligand contact map within 3.5 Å for monastrol at the loop 5 site.

Fig. S4 Temporal protein-ligand contact map within 3.5 Å for compound 1a at the loop 5 site.

Fig. S5 Temporal protein-ligand contact map within 3.5 Å for compound 1a at the loop 8 site.

Fig. S6 Temporal protein-ligand contact map within 3.5 Å for compound 2d at the loop 5 site.

Fig. S7 Temporal protein-ligand contact map within 3.5 Å for compound 2d at the loop 8 site.

Fig. S8 Dynamic cross-correlation map (DCCM) for the Eg5 protein. Positive (red) and negative (blue) coefficients indicate correlated and anticorrelated motions, respectively.

Fig. S9 Dynamic cross-correlation map (DCCM) for monastrol. Positive (red) and negative (blue) coefficients indicate correlated and anticorrelated motions, respectively.

Fig. S10 Dynamic cross-correlation map (DCCM) for compound 1a bound at the loop 5 site. Positive (red) and negative (blue) coefficients indicate correlated and anticorrelated motions, respectively.

Fig. S11 Dynamic cross-correlation map (DCCM) for compound 1a bound at the loop 8 site. Positive (red) and negative (blue) coefficients indicate correlated and anticorrelated motions, respectively.

Fig. S12 Dynamic cross-correlation map (DCCM) for compound 2d bound at the loop 5 site. Positive (red) and negative (blue) coefficients indicate correlated and anticorrelated motions, respectively.

Fig. S13 Dynamic cross-correlation map (DCCM) for compound 2d bound at the loop 8 site. Positive (red) and negative (blue) coefficients indicate correlated and anticorrelated motions, respectively.

# S4. Characterization spectra

Fig. S14 FTIR spectrum (ATR) of compound 1a

Fig. S15 ¹H NMR spectrum of compound 1a (400 MHz, CDCl3)

Fig. S16 ¹³C NMR spectrum of compound 1a (101 MHz, CDCl3)

Fig. S17 MS and MS/MS spectrum of compound 1a

Fig. S18 FTIR spectrum (ATR) of compound 1b

Fig. S19 ¹H NMR spectrum of compound 1b (400 MHz, CDCl3)

Fig. S20 ¹³C NMR spectrum of compound 1b (101 MHz, CDCl3)

Fig. S21 MS and MS/MS spectrum of compound 1b

Fig. S22 FTIR spectrum (ATR) of compound 1c

Fig. S23 ¹H NMR spectrum of compound 1c (400 MHz, CDCl3)

Fig. S24 ¹³C NMR spectrum of compound 1c (101 MHz, CDCl3)

Fig. S25 MS and MS/MS spectrum of compound 1c

Fig. S26 FTIR spectrum (ATR) of compound 1d

Fig. S27 ¹H NMR spectrum of compound 1d (400 MHz, CDCl3)

Fig. S28 ¹³C NMR spectrum of compound 1d (101 MHz, CDCl3)

Fig. S29 MS and MS/MS spectrum of compound 1d

Fig. S30 FTIR spectrum (ATR) of compound 1e

Fig. S31 ¹H NMR spectrum of compound 1e (400 MHz, CDCl3)

Fig. S32 ¹³C NMR spectrum of compound 1e (101 MHz, CDCl3)

Fig. S33 MS and MS/MS spectrum of compound 1e

Fig. S34 FTIR spectrum (ATR) of compound 1f

Fig. S35 ¹H NMR spectrum of compound 1f (400 MHz, CDCl3)

Fig. S36 ¹³C NMR spectrum of compound 1f (101 MHz, CDCl3)

Fig. S37 MS and MS/MS spectrum of compound 1f

Fig. S38 FTIR spectrum (ATR) of compound 2a

Fig. S39 ¹H NMR spectrum of compound 2a (400 MHz, DMSO-d6)

Fig. S40 ¹³C NMR spectrum of compound 2a (101 MHz, DMSO-d6)

Fig. S41 MS and MS/MS spectrum of compound 2a

Fig. S42 FTIR spectrum (ATR) of compound 2b

Fig. S43 ¹H NMR spectrum of compound 2b (400 MHz, DMSO-d6)

Fig. S44 ¹³C NMR spectrum of compound 2b (101 MHz, DMSO-d6)

Fig. S45 MS and MS/MS spectrum of compound 2b

Fig. S46 FTIR spectrum (ATR) of compound 2c

Fig. S47 ¹H NMR spectrum of compound 2c (400 MHz, DMSO-d6)

Fig. S48 ¹³C NMR spectrum of compound 2c (101 MHz, DMSO-d6)

Fig. S49 MS and MS/MS spectrum of compound 2c

Fig. S50 FTIR spectrum (ATR) of compound 2d

Fig. S51 ¹H NMR spectrum of compound 2d (400 MHz, DMSO-d6)

Fig. S52 ¹³C NMR spectrum of compound 2d (101 MHz, DMSO-d6)

Fig. S53 MS and MS/MS spectrum of compound 2d

Fig. S54 FTIR spectrum (ATR) of compound 2e

Fig. S55 ¹H NMR spectrum of compound 2e (400 MHz, CDCl3)

Fig. S56 ¹³C NMR spectrum of compound 2e (101 MHz, CDCl3)

Fig. S57 MS and MS/MS spectrum of compound 2e

# S5. Docking Studies

Fig. S58 2D diagram and hydrophobicity pocket of Monastrol in loop5

Fig. S59 2D diagram and hydrophobicity pocket of compound 1a in loop5

Fig. S60 2D diagram and hydrophobicity pocket of compound 1a in loop8

Fig. S61 2D diagram and hydrophobicity pocket of compound 1b in loop5

Fig. S62 2D diagram and hydrophobicity pocket of compound 1c in loop5

Fig. S63 2D diagram and hydrophobicity pocket of compound 1d in loop8

Fig. S64 2D diagram and hydrophobicity pocket of compound 1e in loop5

Fig. S65 2D diagram and hydrophobicity pocket of compound 1f in loop8

Fig. S66 2D diagram and hydrophobicity pocket of compound 2a in loop5

Fig. S67 2D diagram and hydrophobicity pocket of compound 2b in loop5

Fig. S68 2D diagram and hydrophobicity pocket of compound 2c in loop5

Fig. S69 2D diagram and hydrophobicity pocket of compound 2d in loop5

Fig. S70 2D diagram and hydrophobicity pocket of compound 2d in loop5

# S6. Molecular Dynamics Simulations

Fig. S71 RMSD Analysis

Fig. S72 MM-PBSA Analysis

Fig. S73 RMSF Analysis

Fig. S74 RMSF Analysis Expansion – Cover-Neck Bundle

Fig. S75 RMSF Analysis Expansion – Loop5

Fig. S76 RMSF Analysis Expansion – Loop8

Fig. S77 RMSF Analysis Expansion – Switch I

Fig. S78 RMSF Analysis Expansion – Motor Tip

Fig. S79 RMSF Analysis Expansion – Switch II

Fig. S80 RMSF Analysis Expansion – MT-Binding Site

Fig. S81 RMSF Analysis Expansion – Neck Linker
